# Revisiting the taxonomy and evolution of pathogenicity of the genus *Leptospira* through the prism of genomics

**DOI:** 10.1371/journal.pntd.0007270

**Published:** 2019-05-23

**Authors:** Antony T. Vincent, Olivier Schiettekatte, Cyrille Goarant, Vasantha Kumari Neela, Eve Bernet, Roman Thibeaux, Nabilah Ismail, Mohd Khairul Nizam Mohd Khalid, Fairuz Amran, Toshiyuki Masuzawa, Ryo Nakao, Anissa Amara Korba, Pascale Bourhy, Frederic J. Veyrier, Mathieu Picardeau

**Affiliations:** 1 INRS-Institut Armand-Frappier, Bacterial Symbionts Evolution, Laval, Quebec, Canada; 2 Institut Pasteur, Biology of Spirochetes unit, Paris, France; 3 Université Paris Diderot, Ecole doctorale BioSPC, Paris, France; 4 Institut Pasteur de Nouméa, Leptospirosis Research and Expertise Unit, Nouméa, New Caledonia; 5 Universiti Putra Malaysia, Faculty of Medicine and Health Sciences, Department of Medical Microbiology and Parasitology, Serdang, Malaysia; 6 Universiti Sains Malaysia, Department of Medical Microbiology and Parasitology, Kubang Kerian, Malaysia; 7 Institute for Medical Research, Kuala Lumpur, Malaysia; 8 Chiba Institute of Science, Faculty of Pharmaceutical Sciences, Laboratory of Microbiology and Immunology, Choshi, Japan; 9 Hokkaido University, Department of Disease Control, Graduate School of Veterinary Medicine, Laboratory of Parasitology, Sapporo, Japan; 10 Institut Pasteur d’Alger, Algeria; Instituto Butantan, BRAZIL

## Abstract

The causative agents of leptospirosis are responsible for an emerging zoonotic disease worldwide. One of the major routes of transmission for leptospirosis is the natural environment contaminated with the urine of a wide range of reservoir animals. Soils and surface waters also host a high diversity of non-pathogenic *Leptospira* and species for which the virulence status is not clearly established. The genus *Leptospira* is currently divided into 35 species classified into three phylogenetic clusters, which supposedly correlate with the virulence of the bacteria. In this study, a total of 90 *Leptospira* strains isolated from different environments worldwide including Japan, Malaysia, New Caledonia, Algeria, mainland France, and the island of Mayotte in the Indian Ocean were sequenced. A comparison of average nucleotide identity (ANI) values of genomes of the 90 isolates and representative genomes of known species revealed 30 new *Leptospira* species. These data also supported the existence of two clades and 4 subclades. To avoid classification that strongly implies assumption on the virulence status of the lineages, we called them P1, P2, S1, S2. One of these subclades has not yet been described and is composed of *Leptospira idonii* and 4 novel species that are phylogenetically related to the saprophytes. We then investigated genome diversity and evolutionary relationships among members of the genus *Leptospira* by studying the pangenome and core gene sets. Our data enable the identification of genome features, genes and domains that are important for each subclade, thereby laying the foundation for refining the classification of this complex bacterial genus. We also shed light on atypical genomic features of a group of species that includes the species often associated with human infection, suggesting a specific and ongoing evolution of this group of species that will require more attention. In conclusion, we have uncovered a massive species diversity and revealed a novel subclade in environmental samples collected worldwide and we have redefined the classification of species in the genus. The implication of several new potentially infectious *Leptospira* species for human and animal health remains to be determined but our data also provide new insights into the emergence of virulence in the pathogenic species.

## Author summary

Leptospirosis which is an emerging zoonotic disease worldwide, is transmitted to humans through contact with soils or surface waters contaminated with the urine of reservoir animals. Species of *Leptospira*, which include infectious and non-infectious strains, are ubiquitous in the environment. In this study we have sequenced the genomes of strains of *Leptospira* isolated from several environmental sources worldwide. Comparison of these genomes with other members of the *Leptospira* genus revealed the existence of 30 novel *Leptospira* species. A comparative genomic analysis of species of the genus allowed us to identify genes or genome features that are specific of infectious species, providing insights into virulence evolution in these atypical bacteria but also allow refinement of the *Leptospira* classification.

## Introduction

Leptospirosis is an emerging zoonotic disease of worldwide distribution that affects more than 1 million people with 60,000 deaths per year [1]. In addition, numerous animal hosts (wild and domestic), such as livestock, can contract leptospirosis causing economical cost to subsistence and industrial farming [2]. Exposure to soil or water contaminated with the urine of reservoir animals (mostly rodents) infected with pathogenic *Leptospira* is the most common way in which humans or animals contract leptospirosis [3]. Importantly, the tropism of the infectious agent is not limited to a single host, but rather to multiple hosts that can be asymptomatic carriers or develop mild or severe diseases. The life cycle of pathogenic *Leptospira* is therefore complex, including the natural environment, asymptomatic reservoir and susceptible hosts [4].

Since its original description in 1907 by Stimson [5], the genus *Leptospira* has been traditionally divided into two groups, saprophytes—*Leptospira biflexa sensu lato*—and pathogens—*Leptospira interrogans sensu lato*—based on their virulence. More recently, phylogenetic analysis revealed that *Leptospira* can be divided in three lineages that correlate with the level of pathogenicity of the species: saprophytic, intermediate, and pathogenic [6]. The intermediate species share a near common ancestor with pathogen species while exhibiting moderate pathogenicity in both humans and animals. Both pathogenic and non-infectious environmental saprophytic *Leptospira* strains have been isolated from environmental sources as they are able to survive in moist soil and fresh water for several weeks [7, 8]. The ability of *Leptospira* to occupy various ecological niches is undoubtedly due to a diversity of mechanisms, such as signal transduction systems [9], encoded by its large genome and that allow it to adapt and resist to stressful conditions [9, 10]. It has been suggested that pathogens might have evolved from an environmental ancestor by the acquisition of new functions through lateral gene transfers associated with the adaptation to new hosts [9, 11].

The discovery of novel *Leptospira* species, including species belonging to the pathogen and intermediate lineages, is critical for the development of robust detection and diagnostic tools that are desperately needed to treat infected hosts quicker and adequately. Further characterization of populations of *Leptospira* in soil and water will also help inform prevention and control efforts aimed at reducing the risk of *Leptospira* infection from the environment. It will also enable to better understand the ecology of *Leptospira* in the natural environment and its interactions with other microbial communities. A deeper understanding of the biodiversity of strains that can lead to infections in both humans and animals is lacking. For example, the role of intermediate species in both human and animal infections remains to be clearly established [10]. Information concerning the genetic diversity of circulating *Leptospira* strains is also important to evaluate for the efficacy of current vaccines for the control of leptospirosis. Accurate identification of infectious *Leptospira* is also of prime importance as antibiotic therapy is beneficial in the early stage of the disease.

Isolating *Leptospira* from soil using a novel combination of antimicrobial agents to prevent contamination [12] has recently uncovered many novel *Leptospira* species [13, 14]. The increasing availability of Next-Generation Sequencing (NGS) methods has also provided opportunities to identify novel *Leptospira* species. All together, these recent advances resulted in an important expansion in leptospiral taxonomy with 35 named *Leptospira* species [14, 15].

In the present study, we have isolated new strains from diverse geographical origins and have undertaken a large genomic study in order to dust off the *Leptospira* genus to draw a better picture of its diversity and to propose new standards on its classification and nomenclature to replace the current one that is complex and obsolete [16]. Thus, the classical method of DNA-DNA Hybridization (DDH) for species identification and the serological techniques for serovar identification of *Leptospira* strains will most probably not be used in any laboratory in the near future. The taxonomic status of all species of the genus *Leptospira*, as well as 90 strains isolated from the natural environment across a wide geographic range, was evaluated by comparative genomics. Our results reveal that the genus *Leptospira* now contains 64 named species, including species from a new subclade that is sister to the one that contains the traditional saprophytic species. We propose a new systematic classification scheme of *Leptospira* species to replace the former one that heavily rely on assumption based on virulence level that is often uncharacterized. The high resolution of the dataset used in this study allowed us to investigate the specificities of each clade and to demonstrate significant divergence in pathogenic strains. We have also been able to point a dichotomy in these pathogenic species that is corroborated by different genomic characters. This study will advance many aspects of the leptospirosis field including diagnostics, and basic knowledge including species diversity, evolution, ecology, and virulence.

## Methods

### *Leptospira* strains and culture conditions

*Leptospira* strains used in this study were isolated from water or soil samples from mainland France (two sites), Algeria (one site), Japan (four sites), Malaysia (four sites), Mayotte (four sites) and New Caledonia (three sites) as previously described [14, 17]. *Leptospira* strains were grown at 30°C in liquid Ellinghausen, McCullough, Johnson and Harris (EMJH) medium.

Phenotypic characterization of representative strains was performed by assessing their growth at 14°C, 30°C, and 37°C in liquid EMJH without shaking. Growth in EMJH liquid medium supplemented with 225 μg/ml of the purine analogue 8-azaguanine at 30°C was also tested. Representative strains were plated on 1% agar solid EMJH media and incubated at 30°C until individual subsurface colonies were visible.

Strains used in this study are available at the National Reference Center for Leptospirosis, Institut Pasteur, Paris, France. Type strains of new *Leptospira* species were also deposited in the DSMZ-German Collection of Microorganisms (www.dsmz.de) and the National Collaborating Centre for Reference and Research on Leptospirosis, Amsterdam, The Netherlands (http://leptospira.amc.nl/leptospira-library/), except for species *Leptospira kobayashii*, *Leptospira ryugenii*, *Leptospira ellinghausenii*, and *Leptospira johnsonii* which were deposited in the CIP-Collection of Institut Pasteur (www.pasteur.fr/fr/crbip) and Japan Collection of Microorganisms (http://jcm.brc.riken.jp/en/).

### Ethics statement

Collection of the strains was conducted according to the Declaration of Helsinki. A written informed consent from patients was not required as the study was conducted as part of routine surveillance of the national reference center and no additional clinical specimens were collected for the purpose of the study. Cultures originating from human samples were anonymized. Approval for bacterial isolation from soil and water was not required as the study was conducted as part of investigations of leptospirosis outbreaks. For New Caledonia, approval for bacterial isolation from the natural environment was obtained from the South Province (reference 1689–2017) and North Province (reference 60912-2002-2017).

Protocols for animal experiments conformed to the guidelines of the Animal Care and Use Committees of the Institut Pasteur (Comité d’éthique d’expérimentation animale CETEA # 2016–0019), agreed by the French Ministry of Agriculture. All animal procedures carried out in our study were performed in accordance with the European Union legislation for the protection of animals used for scientific purposes (Directive 2010/63/EU).

### Whole-genome sequencing

In this study, the DNA of a total of 90 *Leptospira* strains were sequenced (S1 Table), including the type strain *L*. *idonii* Eri-1^T^ [13], whose genome sequence was not available. Genomic DNA was prepared by centrifugation of exponential-phase cultures and extraction with MagNA Pure 96 Instrument (Roche). Next-generation sequencing was performed by the Mutualized Platform for Microbiology (P2M) at Institut Pasteur, using the Nextera XT DNA Library Preparation kit (Illumina), the NextSeq 500 sequencing systems (Illumina), and the CLC Genomics Workbench 9 software (Qiagen) for *de novo* assemblies. The draft genomes with 50x minimum coverage were used for subsequent analysis and they were submitted to GenBank; accession numbers are available in S1 Table. The genomic DNA of *L*. *kobayashii* E30^T^, *L*. *ryugenii* YH101^T^, *L*. *ellinghausenii* E18^T^, and *L*. *johnsonii* E8^T^ were sequenced at the Sequencing facility at the University of Hokkaido (Japan) (Mazusawa *et al*. submitted).

### Phylogenetic analyses

A delineation of the species for the genome sequences was performed by Average Nucleotide Identity (ANI) using pyani version 0.2.7 (https://github.com/widdowquinn/pyani). Subsequently, one genome per species was chosen and added to reference genomes available in GenBank, in order to compose a dataset of 64 genomes of *Leptospira*. The genome sequences of *Turneriella parva* DSM 2152 (GenBank Assembly # GCA_000266885.1) and *Leptonema illini* DSM 21528 (GenBank Assembly # GCA_000243335.1) were added as outgroup for phylogenetic analysis. All 66 genomic sequences were annotated with Prokka version 1.12 [18]. The orthology between the coding sequences has been inferred with GET_HOMOLOGUES version 20092018 using the COG and OMCL algorithms [19]. Sequences of 1371 orthologous genes that are in single copy and in the softcore (present in at least 95% of genomes) were codon aligned using MAFFT version 7.397 [20] through TranslatorX version 1.1 [21]. The resulting alignments were filtered using BMGE version 1.12 [22] and concatenated in a partitioned supermatrix using AMAS [23]. The best-fit model was determined for each of the partitions using IQ-TREE version 1.6.7 [24]. A maximum likelihood phylogenetic analysis with 10,000 ultrafast bootstraps was subsequently performed with the same tool [25].

The gene sequences predicted to be in the core genome (present in all genomes) by GET_HOMOLOGUES were aligned as previously described. A phylogenetic tree was made with each of the 553 resulting alignments using IQ-TREE (the best-fit model was found for each of the alignments). The Robinson-Foulds distance was calculated for each of the trees compared to the softcore based one, also using IQ-TREE.

The 16S rRNA sequences of the 66 genomes (including the outgroups), those of strains detected in the environment of the Peruvian Amazon [26] and insectivorous bats from eastern China [27] were aligned and positions of low confidence level masked using SSU-ALIGN version 0.1.1 (http://eddylab.org/software/ssu-align). The best-fit model was determined and a maximum likelihood phylogenetic analysis with 10,000 ultrafast bootstraps was performed with the same tool with IQ-TREE version 1.6.7.

### Genomic analyses

The Amino Acid Identity (AAI) and the Percentage Of Conserved Proteins (POCP) values were determined using GET_HOMOLOGUES version 20092018. The core and pan genome of *Leptospira* was also evaluated using the same tool. The genomic characteristics were determined using a combination of QUAST version 5.0.0 [28], Artemis version 17.0.1 [29] and the DFAST web server (for the number of pseudogenes). The genes coding for lipoproteins have been annotated with SpLip version 1 [30]. Finally, the CDSs were classified into functional categories using eggnog-mapper version 1.0.3 [31] and the PFAM motifs found by InterProScan version 5.31–70.0 [32].

### Statistical analyses

All statistical analyses were performed with PRISM 6. In order to estimate the level of significance between the different clades an Agostino and Pearson omnibus normality test was carried out to check if the data followed a normal distribution. In case where the data were normally distributed, one-way ANOVA with Tukey's multiple test comparisons were performed. In the opposite case, a Kruskal-Wallis test with a Dunn's multiple test comparison were performed. In order to compare the level of significance between the two groups of species composing the S1 subclade (see the result section), normality was also verified by an Agostino & Pearson omnibus normality test. A t-test was then performed if the data were normally distributed or a Mann Whitney test where appropriate.

### Animal experiments

Groups of 4 golden Syrian hamsters (4-week-old males; Janvier, Le Genest, France) were infected via intraperitoneal injection of 10^8^ *L*. *ilyithenensis* and *L*. *ognonensis* type strains or 10^6^ *L*. *interrogans* serovar Manilae strain L495. Animals were monitored daily for clinical signs of leptospirosis (i.e. prostration, jaundice) and survival. Surviving animals were euthanized after a 14 day post-challenge follow-up period, and the kidneys and liver from each animal were harvested for culturing in EMJH medium.

## Results

### Collection of strains and whole-genome sequencing

A collection of environmental isolates from Asia (Japan and Malaysia), Africa (Algeria and the island of Mayotte, a French overseas department in the Indian Ocean), Europe (France) and Oceania (New Caledonia) were included in this study. *Leptospira* isolates were retrieved from water and soil samples from different sites from 2008 to 2017. Except for the Japan isolates [17], environmental isolates reported in this study were not described previously.

The DNA of a total of 90 isolates was sequenced using Illumina technology (S1 Table). The 90 *Leptospira* strains had an average genome size of 4,128,000 ± 221,345 bp. The largest genome was 4,993,538 bp, belonging to *Leptospira putramalaysiae* strain SSW20. The smallest genome was the genome of *Leptospira fletcheri* strain SSW15^T^, 3,733,663 bp in size. The GC content of the genomes in this study ranged from 37.06 to 47.70. The average genome assembly contained 49 ± 50 contigs (S1 Table).

### Phylogenomics and identification of 30 new *Leptospira* species

The complete genome sequences of the 90 strains described in this study were compared to the previously published genome sequences from the already known *Leptospira* species and strain GWTS#1, that was wrongly assigned to the species *Leptospira alstonii* [33, 34] (S1 Table). As a note, we sequenced the genome of *L*. *idonii* strain Eri-1^T^ because a genome sequence was not available for this species [13]. Also, the genome of the recently described species *Leptospira macculloughii* [15] was excluded from further analysis in our list of reference genomes as the genome of *L*. *macculloughii* was the result of a mixed culture of *L*. *meyeri* and *L*. *levetti*.

The results obtained from pairwise comparisons of the 124 genome sequences are summarized as a matrix in the S2 Table. Using a ANI cutoff of 95% generally used as the metrics to delineate bacterial species [35], we established the existence of 64 different species of *Leptospira*. These species include 34 previously described species, 4 new species from Japan (Masuzawa *et al*. submitted), 25 newly isolated species (*Leptospira kemamanensis* sp. nov., *Leptospira andrefontaineae* sp. nov., *Leptospira bandrabouensis* sp. nov., *Leptospira bouyouniensis* sp. nov., *Leptospira congkakensis* sp. nov., *Leptospira dzianensis* sp. nov., *Leptospira dzoumogneensis* sp. nov., *Leptospira fletcheri* sp. nov., *Leptospira fluminis* sp. nov., *Leptospira gomenensis* sp. nov., *Leptospira ilyithenensis* sp. nov., *Leptospira jelokensis* sp. nov., *Leptospira kanakyensis* sp. nov., *Leptospira langatensis* sp. nov., *Leptospira montravelensis* sp. nov., *Leptospira mtsangambouensis* sp. nov., *Leptospira noumeaensis* sp. nov., *Leptospira ognonensis* sp. nov., *Leptospira perdikensis* sp. nov., *Leptospira*. *putramalaysiae* sp. nov., *Leptospira sarikeiensis* sp. nov., *Leptospira selangorensis* sp. nov., *Leptospira semungkisensis* sp. nov., *Leptospira koniamboensis* sp. nov., *Leptospira bourretii* sp. nov.) and one new species (*Leptospira tipperaryensis* sp. nov.) which was previously wrongly assigned to *L*. *alstonii* (strain GWTS#1^T^) based on the 16S rRNA and *secY* genes [33, 34]. Only one representative strain of each of the 64 *Leptospira* species was retained for further analysis. The names and the descriptions of origins of these new species are indicated below. Given our curated database, we assessed the taxonomic assignation of genome sequences already available in GenBank with our corresponding reference strains (S1 File). In doing so, we could easily detect misclassifications of strains (such as for some *L*. *santarosai*, *L*. *weilii* and *L*. *interrogans* assigned strains, see S1 File), which validate the use of our database and the methodology.

### Description of 2 clades and 4 subclades

**Fig 1:**
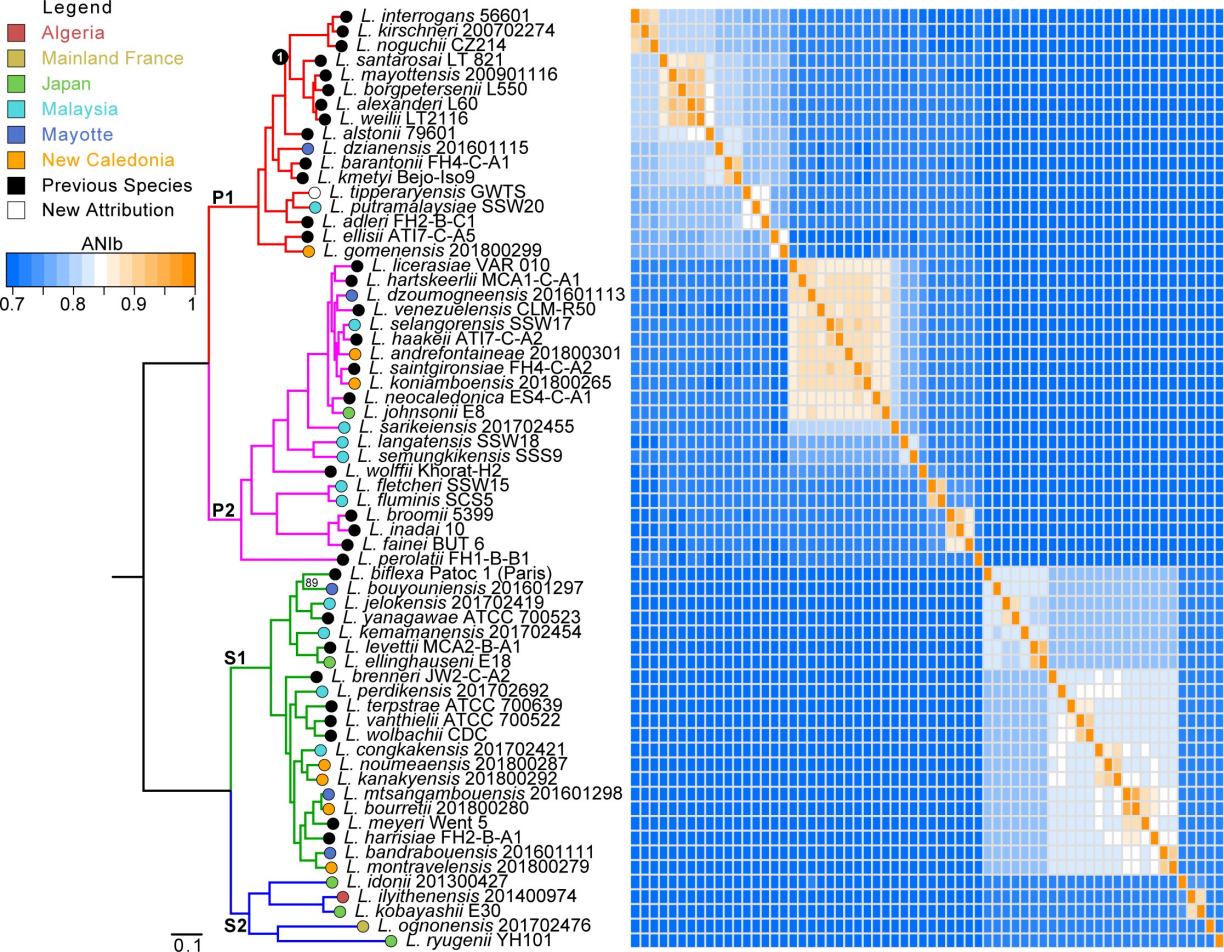
Phylogenetic tree based on the sequences of 1371 genes inferred as orthologous. The matrix represents the calculated ANIb values for all the genomic sequences. The branches are colored according to their belonging to the four main subclades: P1 (red), P2 (purple), S1 (green) and S2 (blue). The bootstrap value is indicated for a single node (that corresponding to the separation between *L*. *biflexa* strain Patoc1 and *L*. *bouyouniensis* strain 201601297) since all the others have the maximum value of 100. A circle of color, according to the legend, represents the geographical origin of each of the new species described by this study. Node 1 indicates the node from which descent pathogenic species most frequently involved in human disease.

The phylogenetic position of each species was robustly evaluated by performing a molecular phylogeny based on 1371 orthologous gene sequences coupled to a matrix of ANI values for each of the 64 *Leptospira* species (Fig 1). The figure confirms 64 well-delineated species of *Leptospira*. The ANI values are consistent with their phylogenetic relationships. The interspecies ANI values ranged from ~69% to ~94%.

Four of the new 30 species, isolated from the natural environment in Malaysia, Mayotte, and New Caledonia and in small mammals in Ireland, were classified within the lineage of pathogens. Ten novel species, isolated from Malaysia, Mayotte, Japan and New Caledonia, were identified as part of the intermediates. Twelve novel species, isolated from Malaysia, Mayotte, Japan, and New Caledonia, were assigned to the saprophytes. Finally, four novel species, isolated from Japan, Algeria, and France, were positioned in a clade sister to the one formed by saprophytes together with *L*. *idonii*. Using this large amount of new species, we could refine the different clades and identified two major clades and four subclades in the *Leptospira* genus. The two major clades are: “Saprophytes” containing species isolated in the natural environment and not responsible for infections and “Pathogens” containing all the species responsible for infections in humans and/or animals, plus environmental species for which the virulence status has not been proven. The two clades are further subdivided in two subclades each. We propose a new nomenclature in order to limit the assumption of virulence character that remain to be characterized (Fig 1): clades P and S and subclade P1 (formerly described as the pathogen group), P2 (formerly described as the intermediate group), S1 (formerly described as the saprophyte group) and S2 (the new subclade described here that includes *L*. *idonii*).

We also compared the amino acid identity (AAI) values from the translated sequences of the different species. This analysis is complementary to that of ANIs in the sense that amino acids evolve less rapidly than nucleotides (degeneracy of the genetic code), thus making it possible to visualize larger and more ancestral groups [36]. As expected, we find the same clades and subclades as with ANI values (S1 Fig). However, the signal is stronger and the clades better defined. Similarly, the percentage of conserved proteins (POCP) values were also calculated across the 64 genome sequences to be compared (S2 Fig). POCP was determined using all the proteins of the genomes to infer the genetic and phenotypic relatedness between a pair of species [37]. With this analysis, we can clearly see the two clades S and P (again confirming the genetic relatedness between the ‘former’ saprophytes clade S1 and the new subclade S2).

### Phenotypic characterization of new species and new subclade S2

The 30 novel *Leptospira* species grow well in liquid EMJH at 30°C. Under dark-field microscopy, strains of these novel species are motile and exhibit the characteristic hook- and spiral-shaped ends that are due to the rotation of the endoflagella. They all exhibit a morphology which is consistent with the genus *Leptospira*, i.e. thin, long and helix-shaped cells.

A more detailed phenotypic analyze of the strains composing the new S2 subclade was performed. *L*. *kobayashii*, *L*. *ilyithenensis*, *L*. *idonii*, and *L*. *ognonensis* were tested for their growth phenotypes under different conditions. Isolates were also selected to provide one representative from each of the three other subclades (P1: *L*. *interrogans* strain L495; P2: *L*. *licerasiae* strain VAR010^T^; S1: *L*. *biflexa* strain Patoc 1). The optimum temperature for growth is 30°C for all *Leptospira* species. The doubling time in liquid EMJH at 30°C is between 15 and 23 hours, except for *L*. *kobayashii* with a doubling time of 8 h. Species of the subclade S2 grow well at 14°C but not at 37°C showing growth characteristics normally observed for species from subclade S1 (formerly called saprophytes) and not observed for species from the P clade that can also grow at 37°C. Similarly, species of the subclade S2 can grow in EMJH supplemented with the purine analogue 8-azaguanine which was usually used as a differential agent for the separation of pathogenic (P1) and saprophytic (S1) *Leptospira* (S4 Table). To evaluate the virulence of species of this new subclade, two representative isolates (*L*. *ognonensis* and *L*. *ilyithenensis*) were injected at high dose (10^8^ bacteria) in hamsters. Animals infected with these novel species did not exhibit any clinical sign of leptospirosis, and bacteria were not recovered from kidneys or livers of infected animals using homogenate’s culture. In contrast, challenge with the virulent *L*. *interrogans* strain L495 caused death in infected hamsters. These results indicate the inability of these novel species to establish acute infection or renal colonization in this animal model.

Cell morphology was similar to those of members of the genus *Leptospira*. Cells were helix-shaped with a length of 9 to 14 μm, a diameter of ~0.2 μm and a wavelength ranging from 0.6 to 0.9 μm (S3 Fig and S4 Table).

Description of *Leptospira dzianensis* sp. nov. : dzi.an.en'sis. N.L. fem. adj. dzianensis of Dziani, a lake in Mayotte. The type strain is M12A^T^, isolated from a water sample in Dziani, Mayotte. Genome Accession Number is RQHR00000000.The genomic G+C content of the type strain is 45.5%. Belonging to the subclade P1.

Description of *Leptospira tipperaryensis* sp. nov. : tip.pe.ra.ry.en'sis. N.L. fem. adj. tipperaryensis pertaining to Tipperary, a county in Ireland. The type strain is GWTS#1^T^, isolated from *Crocidura russula* in Tipperary, Ireland. Previously assigned to *L*. *alstonii* [33, 34], this strain belongs to the subclade P1. Genome Accession Number is GCA_001729245.1. The genomic G+C content of the type strain is 42.4%.

Description of *Leptospira gomenensis* sp. nov. : go.men.en'sis. N.L. fem. adj. gomenensis of Kaala-Gomen, a village in New Caledonia. The type strain is KG8-B22^T^, isolated from a soil sample in Kaala-Gomen, North Province of New Caledonia. Genome Accession Number is RQFA00000000. The genomic G+C content of the type strain is 46.1%. Belonging to the subclade P1.

Description of *Leptospira putramalaysiae* sp. nov. : put.ra.ma.lay'si.ae. N.L. gen. n. putramalaysiae of Putra Malaysia, university hosting the laboratory who isolated the strain. The type strain is SSW20^T^, isolated from a water sample in Sungai Congkak, Malaysia. Genome Accession Number is RQEQ00000000. The genomic G+C content of the type strain is 42.5%. Belonging to the subclade P1.

Description of *Leptospira andrefontaineae* sp. nov. : an.dre.fon.tai'ne.ae. N.L. gen. n.andrefontaineae of André‐Fontaine, named in honor of Geneviève André‐Fontaine, a french veterinarian, who made significant contribution to the study of animal leptospirosis. The type strain is PZF11-2^T^, isolated from a water sample in Nouméa, New Caledonia. Genome Accession Number is RQEY00000000. The genomic G+C content of the type strain is 39.9%. Belonging to the subclade P2.

Description of *Leptospira dzoumogneensis* sp. nov. : dzou.mog.ne. en'sis. N.L. fem. adj. dzoumognensis of Dzoumogné, a village in Mayotte. The type strain is M11A^T^, isolated from a water sample in Dzoumogné, Mayotte. Genome Accession Number is RQHS00000000. The genomic G+C content of the type strain is 41.0%. Belonging to the subclade P2.

Description of *Leptospira koniamboensis* sp. nov. : N.L. fem. adj. koniamboensis of Koniambo, mountain in New Caledonia. The type strain is TK1-4^T^, isolated from a water sample in Koné, North Province of New Caledonia. Genome Accession Number is RQFY00000000. The genomic G+C content of the type strain is 39.0%. Belonging to the subclade P2.

Description of *Leptospira sarikeiensis* sp. nov. : sa.ri.kei.en'sis. N.L. fem. adj. sarikeienis of the district of Sarikei. The type strain is LIMR175^T^, isolated from a water sample in Sarawak, Malaysia. Genome Accession Number is RQGF00000000. The genomic G+C content of the type strain is 40.3%. Belonging to the subclade P2.

Description of *Leptospira johnsonii* sp. nov. : john.so'ni.i. N.L. gen. n. johnsonii of Johnson, named in honor of Russel C. Johnson, an American microbiologist who developed EMJH medium that is commonly used for *Leptospira* culture. The type strain is E8^T^, isolated from a soil sample in Ibaraki, Japan [17]. Genome Accession Number is BFAY00000000. The genomic G+C content of the type strain is 41.3%. Belonging to the subclade P2.

Description of *Leptospira fluminis* sp. nov. : flu'mi.nis. L. gen. n. fluminis of a river. The type strain is SCS5^T^, isolated from a soil sample in Sungai Congkak, Malaysia. Genome Accession Number is RQEV00000000.The genomic G+C content of the type strain is 47.7%. Belonging to the subclade P2.

Description of *Leptospira fletcheri* sp. nov. : flet'che.ri. N.L. gen. n. fletcheri of Fletcher, named in honor of William Fletcher who reported the first case of leptospirosis in Malaysia in 1927. The type strain is SSW15^T^, isolated from a water sample in Sungai Congkak, Malaysia. Genome Accession Number is RQET00000000. The genomic G+C content of the type strain is 47.3%. Belonging to the subclade P2.

Description of *Leptospira semungkisensis* sp. nov. : se.mung.kis.en'sis. N.L. fem. adj. semungkisensis of Semungkis, a river in the Hulu Langat district of Selangor state, Malaysia. The type strain is SSS9^T^, isolated from a soil sample in Sungai Congkak, Malaysia. Genome Accession Number is RQEP00000000. The genomic G+C content of the type strain is 42.8%. Belonging to the subclade P2.

Description of *Leptospira langatensis* sp. nov. : lan.gat.en'sis. N.L. fem. adj. langatensis of the district of Langat, Malaysia. The type strain is SSW18^T^, isolated from a water sample in Sungai Congkak, Malaysia. Genome Accession Number is RQER00000000. The genomic G+C content of the type strain is 44.8%. Belonging to the subclade P2.

Description of *Leptospira selangorensis* sp. nov. : se.lan.gor.en'sis. N.L. fem. adj. selangorensis of the state of Selangor, Malaysia. The type strain is SSW17^T^, isolated from a water sample in Sungai Congkak, Malaysia. Genome Accession Number is RQES00000000. The genomic G+C content of the type strain is 40.0%. Belonging to the subclade P2.

Description of *Leptospira ognonensis* sp. nov. : og.non.en'sis. N.L. fem. adj. ognonensis of Ognon, river in France. The type strain is 201702476^T^, isolated from a water sample in the region Bourgogne-Franche-Comté, France. Genome Accession Number is RQHS00000000. The genomic G+C content of the type strain is 39.7%. Belonging to the subclade S2.

Description of *Leptospira ilyithenensis* sp. nov. : il.yi.then.en'sis. N.L. fem. adj. ilyithenensis of Ilyithen, a village located in Algeria. The type strain is 201400974^T^, isolated from a water sample in Ilyithen, a village located in the Djurdjura mountains, Algeria. Genome Accession Number is RQHV00000000. The genomic G+C content of the type strain is 40.5%. Belonging to the subclade S2.

Description of *Leptospira kobayashii* sp. nov. : ko.ba.ya'shi.i. N.L. gen. n. kobayashii of Kobayashi, named in honor of Yuzuru Kobayashi, a Japanese physician and microbiologist who introduced monoclonal antibodies for the classification of *Leptospira*. The type strain is E30^T^, isolated from a soil sample in Gifu, Japan [17]. Genome Accession Number is BFBA00000000. The genomic G+C content of the type strain is 40.7%. Belonging to the subclade S2.

Description of *Leptospira ryugenii* sp. nov. : ru.ge'ni.i. N.L. gen. n. ryugenii of Ryugen, nicknamed in honor of Yasutake Yanagihara, a Japanese microbiologist, University of Shizuoka, who contributed to the chemotaxonomy and study of *Leptospira*. The type strain is YH101^T^, isolated from a water sample in Shizuoka, Japan [17]. Genome Accession Number is BFBB00000000. The genomic G+C content of the type strain is 39.9%. Belonging to the subclade S2.

Description of *Leptospira bandrabouensis* sp. nov. : ban.dra.bou.a.en'sis. N.L. fem. adj. bandrabouaensis of Bandraboua, a commune in Mayotte. The type strain is M10A^T^, isolated from a water sample in Bandraboua, Mayotte. Genome Accession Number is RQHT00000000. The genomic G+C content of the type strain is 37.9%. Belonging to the subclade S1.

Description of *Leptospira noumeaensis* sp. nov. : nou.me.a.en'sis. N.L. fem. adj. noumeaensis of Nouméa, the capital city of New Caledonia. The type strain is PZF14-4^T^, isolated from a water sample in Nouméa, South Province of New Caledonia. Genome Accession Number is RQFK00000000. The genomic G+C content of the type strain is 38.3%. Belonging to the subclade S1.

Description of *Leptospira jelokensis* sp. nov. : je.lok.en'sis. N.L. fem. adj. Jelokensis of Jelok, a housing area in Kajang from where the strain was isolated. The type strain is L5S1^T^, isolated from a soil sample in Sungai Jelok, Malaysia. Genome Accession Number is RQGR00000000. The genomic G+C content of the type strain is 38.9%. Belonging to the subclade S1.

Description of *Leptospira bourretii* sp. nov. : N.L. gen. n. bourretii of Bourret, named in honor of Henri Désiré Gaston Bourret (1875–1917), a medical doctor and microbiologist who developed medical microbiology in New Caledonia. The type strain is PZF7-6^T^, isolated from a soil sample in Nouméa, South Province of New Caledonia. Genome Accession Number is RQFM00000000. The genomic G+C content of the type strain is 38.2%. Belonging to the subclade S1.

Description of *Leptospira kanakyensis* sp. nov. : ka.na.ky.en'sis. N.L. fem. adj. kanakyensis of Kanaky, the name of "New Caledonia" for Kanak people. The type strain is TK5-11^T^, isolated from a soil sample in Koné, North Province of New Caledonia. Genome Accession Number is RQFG000000. The genomic G+C content of the type strain is 38.5%. Belonging to the subclade S1

Description of *Leptospira kemamanensis* sp. nov. : ke.ma.man.en'sis. N.L. fem. adj. kemamanensis of the district of Kemaman. The type strain is LIMR131^T^, isolated from a water sample in Terengganu, Malaysia. Genome Accession Number is RQGG000000. The genomic G+C content of the type strain is 38.9%. Belonging to the subclade S1.

Description of *Leptospira mtsangambouensis* sp. nov. : mtsan.gam.bou.en'sis. N.L. fem. adj. mtsangambouensis of Mtsangamboua, a village in Mayotte. The type strain is M2A^T^, isolated from a water sample in Mtsangamboua, Mayotte. Genome Accession Number is RQHK00000000. The genomic G+C content of the type strain is 38.2%. Belonging to the subclade S1.

Description of *Leptospira bouyouniensis* sp. nov. : bou.you.ni.en'sis. N.L. fem. adj. bouyouniensis of Bouyouni, a village in Mayotte. The type strain is M1A^T^, isolated from a water sample in Bouyouni, Mayotte. Genome Accession Number is RQHL00000000. The genomic G+C content of the type strain is 37.1%. Belonging to the subclade S1.

Description of *Leptospira ellinghausenii* sp. nov. : el.ling.hau.se'ni.i. N.L. gen. n. ellinghausenii of Ellinghausen, named in honor of Herman C. Ellinghausen, an American microbiologist who developed the EMJH medium that is commonly used for the culture of *Leptospira*. The type strain is E18^T^, isolated from a soil sample in Fukushima, Japan [17]. Genome Accession Number is BFAZ00000000. The genomic G+C content of the type strain is 37.3%. Belonging to the subclade S1.

Description of *Leptospira congkakensis* sp. nov. : cong.kak.en'sis. N.L. fem. adj. congkakensis of Congkak, a recreational forest in the Hulu Langat district of Selangor state, Malaysia. The type strain is SCS9^T^, isolated from a soil sample in Sungai Congkak, Malaysia. Genome Accession Number is RQGQ00000000. The genomic G+C content of the type strain is 38.2%. Belonging to the subclade S1.

Description of *Leptospira perdikensis* sp. nov. : per.dik.en'sis. N.L. fem. adj. perdikensis of Perdik, name of the waterfall where the strain was isolated. The type strain is HP2^T^, isolated from a water sample in Hulu Perdik, Malaysia. Genome Accession Number is RQGA00000000. The genomic G+C content of the type strain is 38.5%. Belonging to the subclade S1.

Description of *Leptospira montravelensis* sp. nov. : mont.ra'vel.en'sis. N.L. fem adj. montravelensis of Montravel, the district in Nouméa where the type strain PZF5-3 ^T^ was isolated, itself named from the French navigator Tardy de Montravel (1811–1864) who contributed to the cartography of New Caledonia. Genome Accession Number is RQFN00000000. The genomic G+C content of the type strain is 37.4%. Belonging to the subclade S1.

### Genomic characteristics of the four distinct subclades

**Fig 2:**
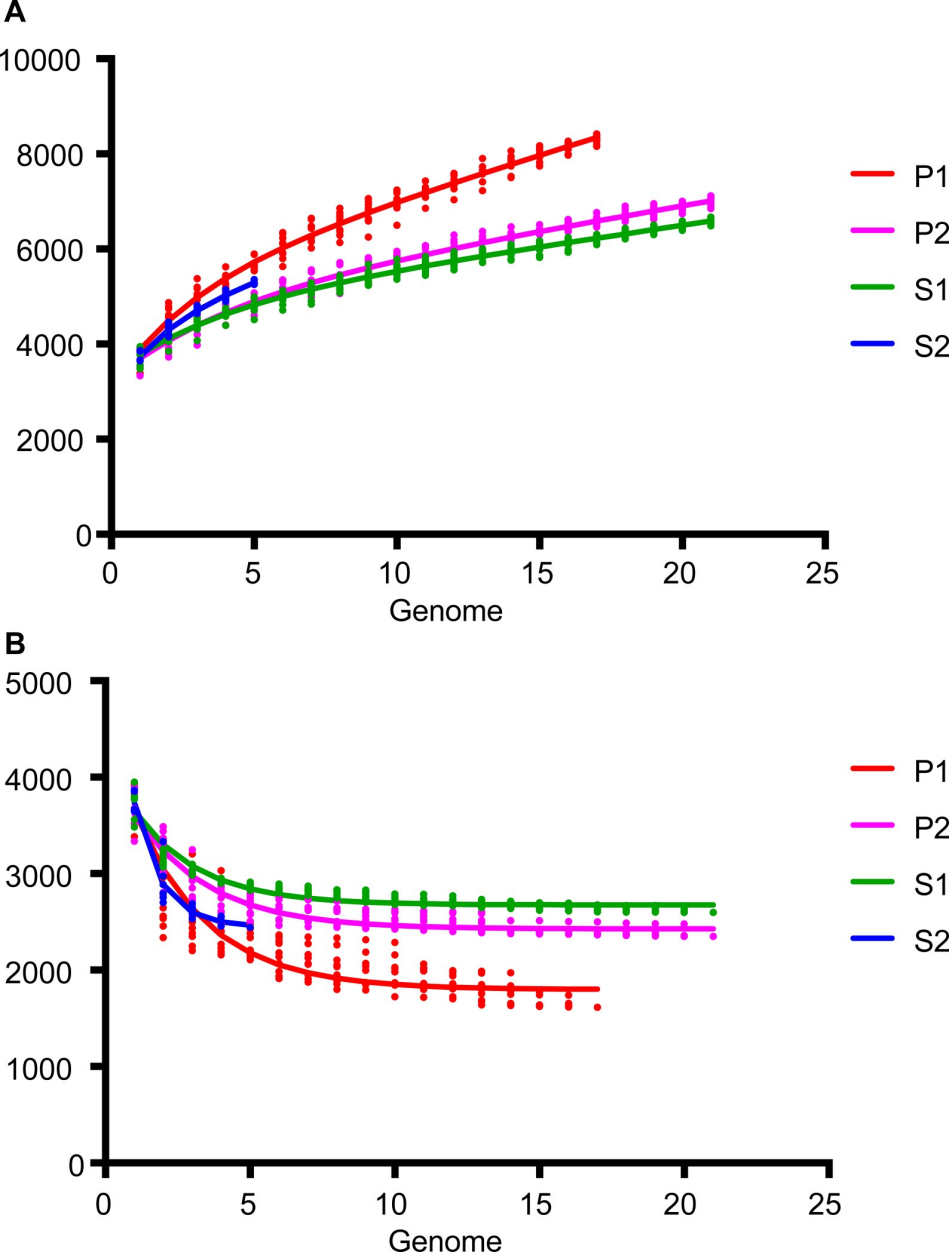
**Evolution of (A) pan-genome and (B) core-genome for the genomes of the four leptospiral subclades.** Analyses done with GET_HOMOLOGUES (using 17 genomes for P1, 21 for P2, 21 for S1 and 5 for S2) highlighting that the P1 group has a more open pan-genome.

**Fig 3:**
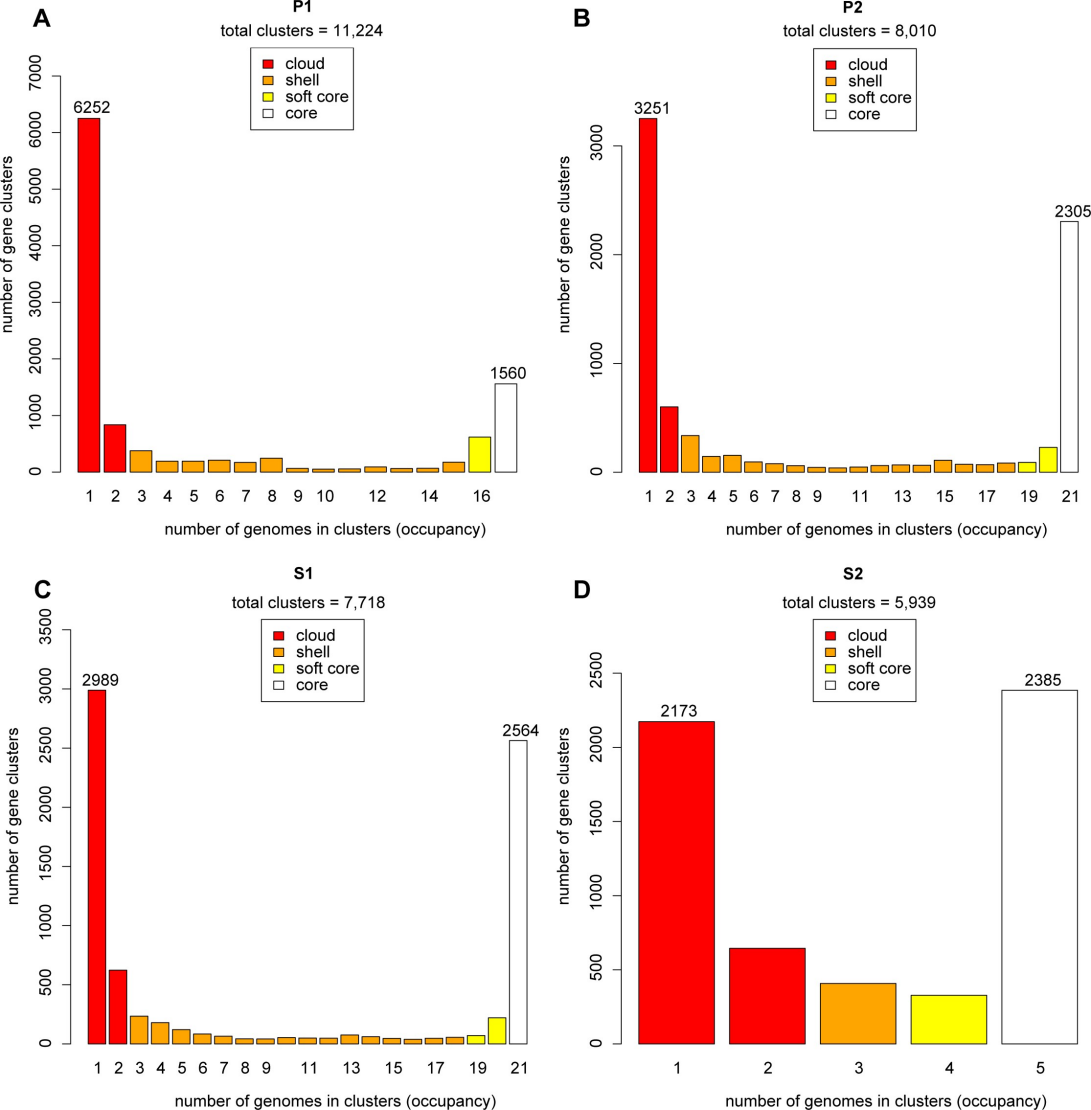
**Pan-genome distribution in four categories (cloud, shell, soft core and core) for species from subclades (A) P1, (B) P2, (C) S1 and (D) S2.** Analyses done with GET_HOMOLOGUES (using 17 genomes for P1, 21 for P2, 21 for S1 and 5 for S2) showing the U-shaped distribution of pan-genome from the four groups. However, strains of the P1 group show asymmetry by having four times more single species than core genes.

In addition, knowing that there are four distinct phylogenetic subclades of *Leptospira* species, it was interesting to verify the opening of the pan-genome between and within each of the subclades (Fig 2). Not surprisingly, the subclades are distinct from each other in this analysis. Interestingly, the P1 subclade harbors the most open pan-genome. This suggests a great diversity in the gene repertoire in this specific subclade. The fact that the P1 has a more open pan-genome has led us to verify the gene distribution for the different groups (Fig 3). This analysis shows that, unlike species from P1, all groups have approximately the same number (≈1X) of genes cluster in the core genome (an orthologous gene in all genomes) compared to gene clusters found in one species (gene present in only one species). Within the P1 subclade, there is a strong enrichment (≈4X) of gene clusters that are unique to one species (6252) as compared to gene clusters in the core genome (1560).

**Fig 4:**
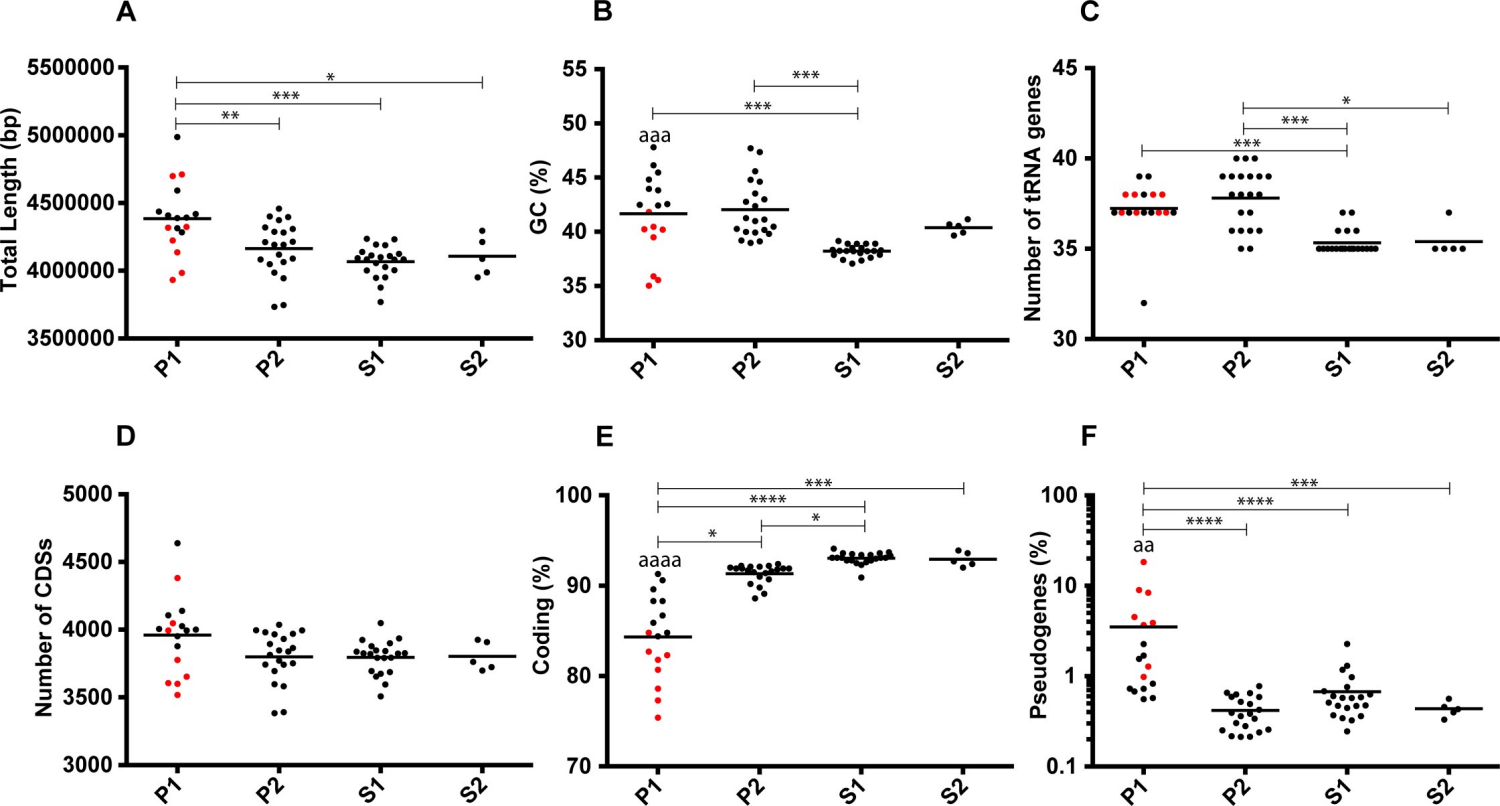
**Distribution of (A) total length, (B) GC %, (C) number of tRNA genes, (D) number of CDSs, (E) coding % and (F) pseudogenes % (values in log) in the four major subclades.** The points representing the genome-specific values of the species that diverged after node 1 in Fig 1 (*L*. *interrogans*, *L*. *kirschneri*, *L*. *noguchii*, *L*. *santarosai*, *L*. *mayottensis*, *L*. *borgpetersenii*, *L*. *alexanderi* and *L*. *weilii*) are in red. The "*" represent the level of significance between the different groups: * P ≤ 0.05, ** P ≤ 0.01, *** P ≤ 0.001, and **** P ≤ 0.0001. The level of significance between the two P1 groups separated by node 1 is represented by the same code, but for the sake of clarity the symbol is "a".

To better define the genomic characteristics of the four subclades, we compared different general features as shown in Fig 4. Members of the subclade P1 are often significantly more divergent than those composing the other subclades. In general, genomes of species belonging to P1 tend to be larger, have a higher and scattered GC content (common to P2), harbor more genes encoding tRNAs (common to P2), have a lower coding ratio, and a higher number of pseudogenes. For several of the features investigated, the subclade P1 presented a scattered distribution. Other studies have already noticed the presence of subgroups of species and unusual discrepancies in some genomic characteristics [11, 15]. To verify if this could be seen in our analyses we separated the subclade P1 into two groups with one group being the species that diverged after a specific node of evolution (node 1 in Fig 1 that separates species frequently involved in infections). Both groups are effectively significantly divergent for the GC %, the coding ratio and the percentage of pseudogenes.

**Fig 5:**
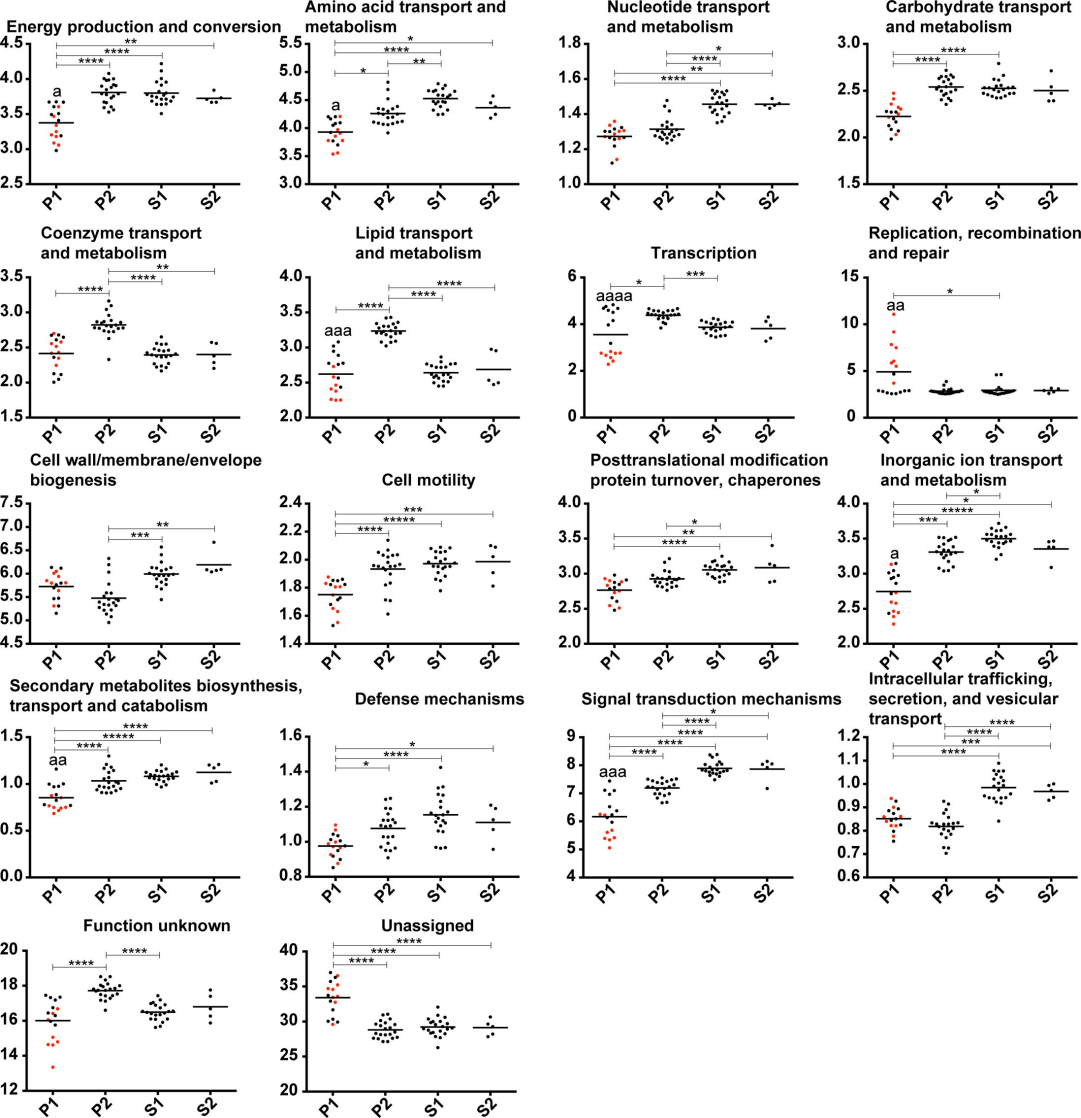
Distribution in functional categories of the predicted CDSs (%). The points representing the genome-specific values of the species that diverged after node 1 in Fig 1 (*L*. *interrogans*, *L*. *kirschneri*, *L*. *noguchii*, *L*. *santarosai*, *L*. *mayottensis*, *L*. *borgpetersenii*, *L*. *alexanderi* and *L*. *weilii*) are in red. The "*" represent the level of significance between the different groups: * P ≤ 0.05, ** P ≤ 0.01, *** P ≤ 0.001, and **** P ≤ 0.0001. The level of significance between the two pathogenic groups is represented by the same code, but for the sake of clarity the symbol is "a". Only functional categories showing significant difference are shown.

To further characterize the subclades, the CDSs of the different genomes were grouped into functional categories to assess potential enrichments in one of them (Fig 5). A total of 16 categories involved in known functions are significantly enriched in at least one subclade (p < 0.05). The two groups of the subclade P1 can also be significantly separated for eight out of the 16 categories. It is interesting to note that around a third of the CDSs were not assigned to functional categories for all subclades and that species belonging to the subclade P1 harbor significantly more unassigned CDSs. These proteins have no similarity with functional categories including COGs with unknown function. This may represent remnants of pseudogenes, wrong annotation, as well as proteins restricted to the *Leptospira* genus.

**Fig 6:**
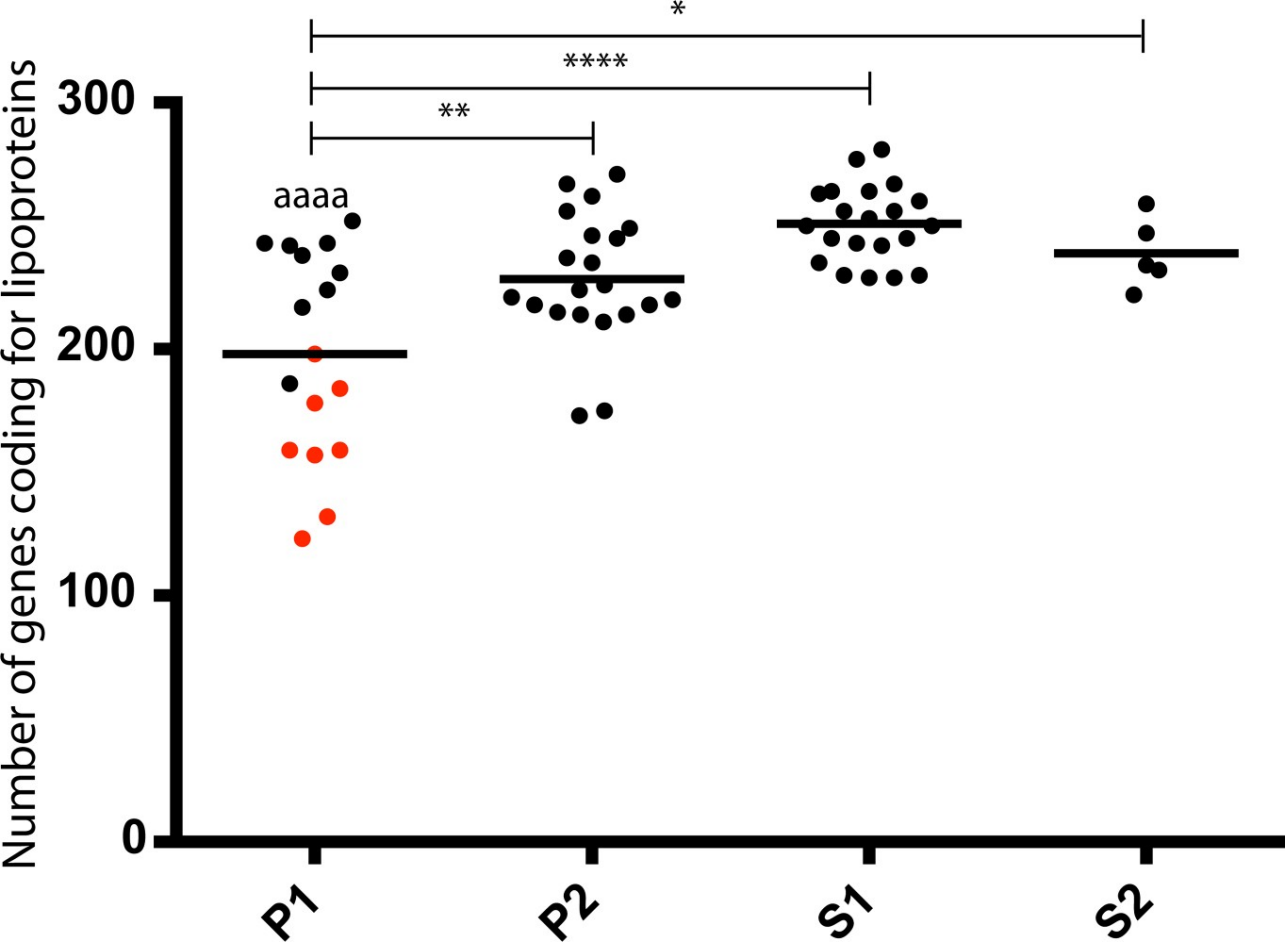
Distribution of genes encoding lipoproteins. The "*" represent the level of significance between the different groups: * P ≤ 0.05, ** P ≤ 0.01, *** P ≤ 0.001, and **** P ≤ 0.0001. The level of significance between the two pathogenic P1 groups (before and after node 1) is represented by the same code, but for the sake of clarity the symbol is "a". The points representing the genome-specific values of the species that diverged after node 1 in Fig 1 (*L. interrogans, L. kirschneri, L. noguchii, L. santarosai, L. mayottensis, L. borgpetersenii, L. alexanderi* and *L. weilii*) are in red.

**Fig 7:**
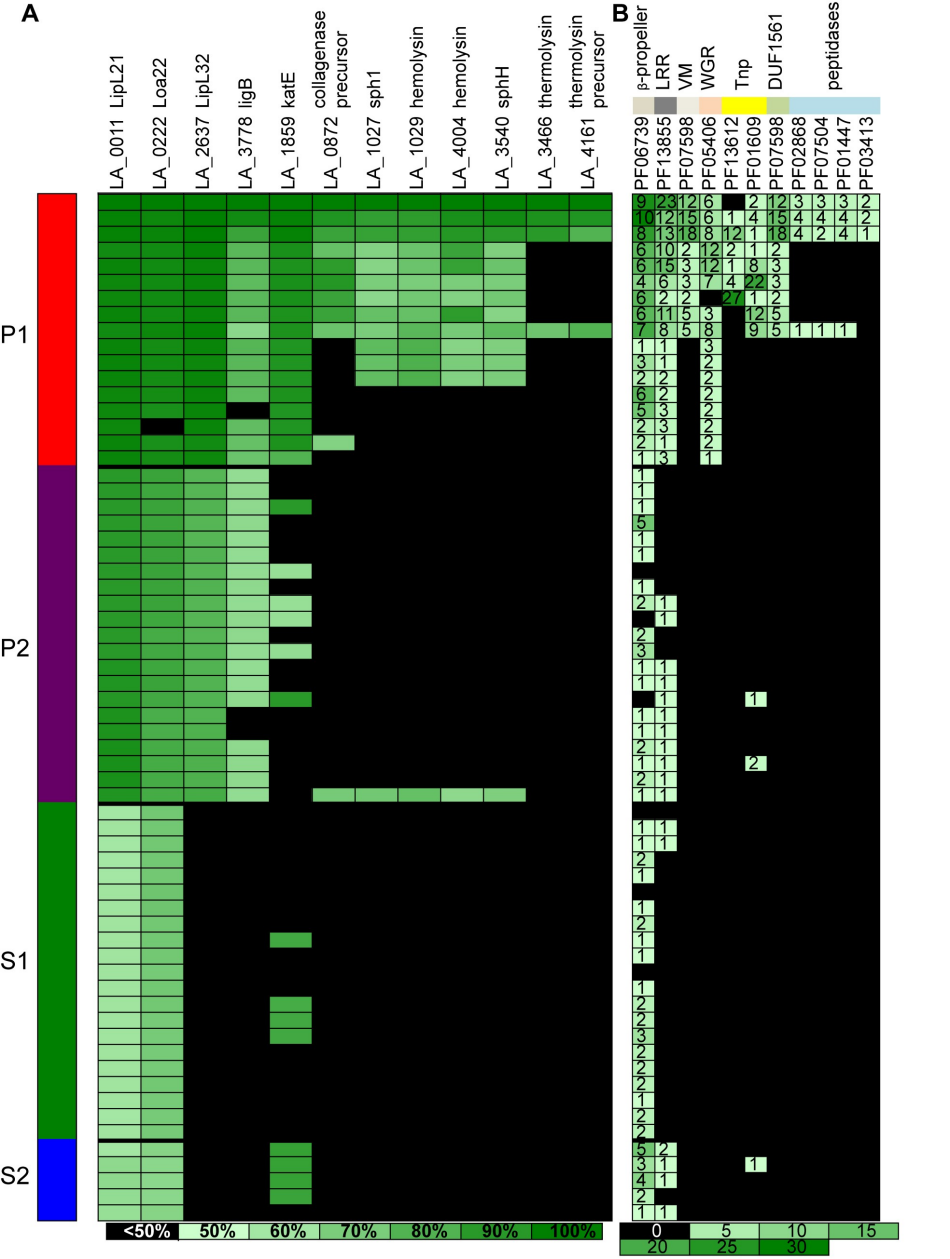
**Distribution of genes involved in virulence (A) and PFAM motifs (B).** The gradient represents for (A) the percentage of similarity according to the homologous proteins sequences in *L*. *interrogans* strain 56601 and for (B) the number of genes having the different PFAM motifs.

Finally, we investigated the number of lipoproteins and the distribution of known virulence factors from the updated genus (Figs 6 and 7). Interestingly, lipoproteins, which are membrane proteins, are coded by a lower number of genes in the P1 subclade, in comparison to the other subclades; this is particularly true for the species that diverged after node 1 (most virulent species) within the P1 subclade (*L*. *interrogans*, *L*. *kirschneri*, *L*. *noguchii*, *L*. *santarosai*, *L*. *mayottensis*, *L*. *borgpetersenii*, *L*. *alexanderi* and *L*. *weilii*) (Fig 6). In contrast, as expected, it is possible to observe a gradient in the repertoire of genes encoding proteins known to be involved in virulence (Fig 7A). The species of subclades P1 and P2 having the most genes encoding virulence factors and S1 and S2 having the least genes. However, it is interesting to note that the distribution of the gene coding for KatE catalase (LA1859), that is an important virulence factor in animal model [38], is more heterogeneous than previously suspected [10], as it is possible to confidently find an homologous copy in genomes of some strains belonging to subclades P2, S1 and S2. Several PFAM domains are known to be associated with proteins involved in *Leptospira* virulence [10]. As expected, it has been possible to find a much larger number of these domains in the P1 species, more particularly in the species that diverged after node 1 with a high level of pathogenicity in humans (Fig 7B).

### 16S rRNA data is insufficient to robustly distinguish *Leptospira* species

**Fig 8:**
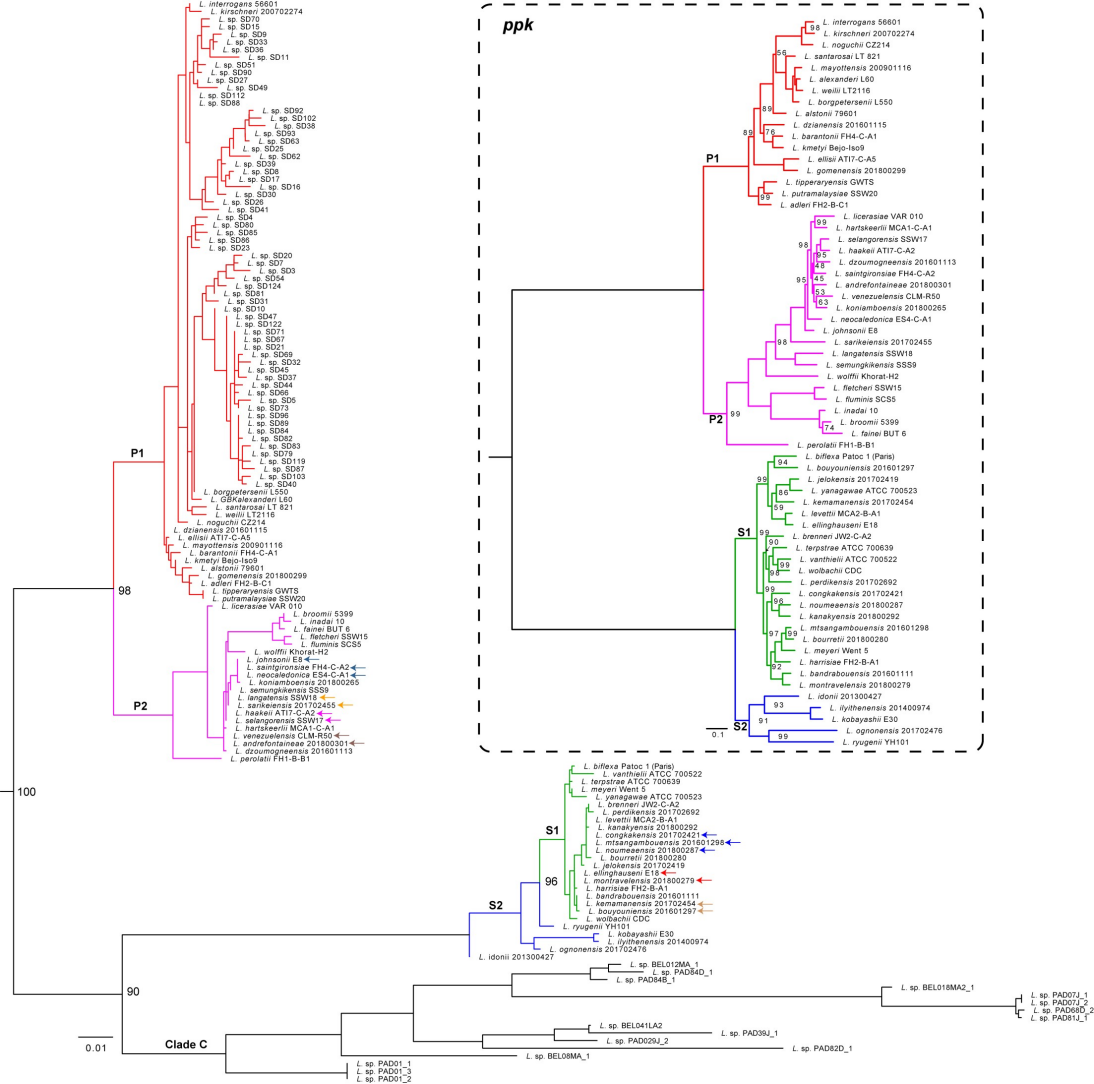
Phylogenetic tree based on the 16S rRNA and *ppk* sequences to evaluate the diversity within the *Leptospira* genus. In addition to the 16S rRNA sequences from the 64 genomes investigated in the present study, those from uncultured strains from the Peruvian Amazon (Clade C) [26] and from insectivorous bats from eastern China [27] were added. The branches are colored according to their belonging to the four main subclades: P1 (red), P2 (purple), S1 (green) and S2 (blue), while the strains of the “clade C” are in black. For the sake of clarity, the bootstrap values are only indicated for the nodes that correspond to the major splits. A tree constructed with the *ppk* gene sequences is included in the dashed box for comparison. In this case, all bootstrap values less than 100 are indicated at the different nodes.

Phylogenetic reconstruction based on 16S rRNA gene sequences is a widely used approach to infer relationships between bacteria. Nevertheless, the high conservation of rRNA reduces its discriminatory power and 16S rRNA sequences may not be sufficient to distinguish related bacterial species. In the light of the robustly updated genus, we investigated the power of resolution of the 16S rRNA sequences for *Leptospira*. We found that i) *L*. *johnsonii*, *L*. *saintgironsiae* and *L*. *neocaledonica*, ii) *L*. *langatensis* and *L*. *sarikeiensis*, iii) *L*. *haakeii* and *L*. *selangorensis*, iv) *L*. *venezuelensis* and *L*. *andrefontaineae*, v) *L*. *congkakensis*, *L*. *mtsangambouensis* and *L*. *noumeaensis*, vi) *L*. *ellinghausenii* and *L*. *montravelensis*, and vii) *L*. *kemamanensis* and *L*. *bouyouniensis* have 100% identical 16S rRNA sequences (Fig 8). A phylogenetic analysis with these sequences and others available in GenBank permitted to recover the separation of the species into four large subclades P1, P2, S1 and S2 (Fig 8). Although less resolutive than the phylogenetic analysis with softcore genes, 16S rRNA analysis allows to appreciate the potential diversity that remains to be explored in *Leptospira*. In this sense, sequences from bats from China [27] clustered among P1 and long lengths of branches of some subclades suggest that some of these strains could correspond to unknown, potentially novel species yet to be isolated. A striking result was the high diversity of sequences recovered from the environment of the Peruvian Amazon and composing the previously named “clade C” [26]. The “clade C” is predicted to be sister to the S clade (Fig 8).

We searched among the genes of the core genome which would allow to obtain a topology closest to that inferred with all softcore genes. A total of 553 phylogenetic trees (from the 553 genes of the core genome in single copy) were compared to the softcore tree. We found that the *ppk* gene (LA3459 in *L*. *interrogans*), encoding a polyphosphate kinase of 712 aa in *L*. *interrogans*, made it possible to reproducibly obtain the tree with the lowest Robinson-Foulds distance. The tree generated from the sequences of the *ppk* gene effectively makes it possible to recover the monophyly of the four subclades (P1, P2, S1 and S2) (Fig 8).

## Discussion

In this study, 90 genomes of *Leptospira* strains collected from soil and water samples from 18 different sites across four continents were sequenced. The genome relatedness between these environmental isolates and representative strains of each of the known species of *Leptospira* allowed us to identify 30 new species. We propose to reclassify species of the *Leptospira* genus into 4 subclades, called P1, P2, S1 and S2, instead of the clusters historically named as saprophytes (S1 and S2), intermediates (P2) and pathogens (P1).

Traditionally, classification of bacteria is performed on the basis of their phenotypic characteristics, such as Gram staining, growth requirements, and biochemical tests. Low phenotypic diversity within the *Leptospira* genus precludes from using differential growth characteristics for differentiation of *Leptospira* at the species level. Only a few phenotypic tests such as virulence in animal models, growth rate at 30°C, growth at 37°C or 14°C and growth in the presence of the purine analogue 8-azaguanine can be used to separate the P1 (former pathogens) from S1 (former saprophytes). Modern microbial taxonomy is primarily based on 16S rRNA gene relationships, enabling strain identification at the level of species in most cases. The 16S phylogenetic analysis of the present study, while allowing visualizing the general diversity of the genus *Leptospira*, shows the weakness of this gene to make a robust and precise phylogenetic inference. For example, it was impossible to find the monophyly of subclade S2 with respect to S1. The 16S sequences are often highly conserved, and therefore often lack sufficient variable characters to make a robust phylogenetic inference at the species level [10]. In addition, the two copies of the 16S gene in *Leptospira* genomes may be divergent and come from horizontal transfers, and thus bias phylogenetic reconstruction (reviewed in [10]). The ability to achieve robust phylogenetic classification from a single gene is, however, important in a diagnostic context where it may be unrealistic to effectively and rapidly perform a phylogeny based on several hundred genes. We therefore looked for a candidate gene among the core genome. It turned out that the *ppk* gene, encoding a polyphosphate kinase, makes it possible to reproducibly recover a topology very similar to that obtained from softcore genes (Fig 8). Previous studies in other bacteria, such as "*Candidatus* Accumulibacter" [39] and *Microbacterium* [40], have shown that the *ppk* gene evolves rapidly, allowing phylogenetic reconstructions.

The advent of high-throughput DNA sequencing has changed our view of bacterial taxonomy. This is particularly true for fastidious bacteria such as *Leptospira*. With the increase in available sequences, genome-wide comparisons can be highly discriminative allowing precise taxonomic classification. Among the various *in silico* genome-to-genome comparison methods studied, the ANI, AAI, and POCP values were shown to yield good correlation with phylogenetic studies and the traditional DNA-DNA hybridization values [35, 37, 41, 42]. If possible, suspected new species genome must be sequenced and ANI could be calculated to our curated species database publicly available (http://fveyrier.profs.inrs.ca/Download/Dataset.zip). This method clearly avoids misidentification of species (as demonstrated in this study by using the NCBI public database) and can enable the identification of new species (cut-off >95%). The use of ANI values can also delineate some clear subgroups within the four subclades. Interestingly enough, the subclades P1 and P2 seem to be constituted by multiple small subgroups, representing a high level of diversity. As a note the segmentation of the subclade P1 in groups have been already described [9, 11, 15, 43]. Also, the species forming the new subclade S2 are clearly among the most diverse in ANI values, consistent with the long branches in the phylogenetic tree.

Our study identified a total of 64 species with four new species (*L*. *gomenensis*, *L*. *putramalaysiae*, and *L*. *dzianiensis*, *L*. *tipperaryensis*) in the P1 subclade. We also identified ten new species in subclade P2 (old “intermediate” group). Finally, sixteen new *Leptospira* species isolated from the natural environment belonged to subclades S1 and S2. We showed that species of the new subclade S2 possess phenotypic characteristics of saprophytes S1, which is consistent with their phylogenetic position. *Leptospira* species are considered ubiquitous, as they are found in a wide variety of environments including surface water, soil, and they are found in mammals but also in birds, amphibians, and reptiles [2, 44, 45]. Recent isolation of 12 novel species from tropical soils in areas of endemic leptospirosis in New Caledonia suggests that soils are an important niche for the genus [14, 15]. Our study, where we collected soil and water samples from a wide range of ecosystem types (tropical forests, temperate and Mediterranean freshwaters) worldwide, further supports that this genus is highly diverse and *Leptospira* spp. are found in abundance in both soil and water throughout the different continents. Among the sequenced environmental isolates, several saprophytic species were found onto different continents. For example, *L*. *meyeri* was isolated in France, New Caledonia, and Malaysia; *L*. *bandrabouensis* in Mayotte and New Caledonia (S3 Table). The mechanisms of dispersion of these non-pathogenic species with no know animal reservoirs remain to be determined, especially in the context of tropical islands.

The evolution of the *Leptospira* genus is still puzzling. The current hypothesis is that *Leptospira* genus is broadly found in soil and water and that symbiosis of leptospires, including commensals or pathogens, with eukaryotes emerged from free-living ancestral species in a stepwise and independent manner, as suggested by different accessory genes [15]. The genomic analyzes presented in the present study allow a better understanding of the evolution of the species forming the different clades and subclades. An open pan-genome is typical of bacteria living sympatrically with other species and with a high rate of horizontal gene transfer [46], a feature of soil microbiota. It was already known that the genus has an open pan-genome [9]. With more species, we were able to refine this pan-genome in the different subclades and demonstrated that the P1 subclade has the most open pan genome. This result is corroborated by the fact that the pan-genome distribution of species belonging to P1 clade is asymmetrically U-shaped, with many genes specifically found in single species. This suggests a massive reworking of the cellular functions in this subclade by multiple horizontal gene transfers that could have allowed a change in the ecological niche occupied from a free-living to non-obligatory symbiotic (commensal or pathogen) organism. This correlates with a generally larger genome of species from the P1 subclade. Although the reason is not yet completely clear, it is possible to think that the large range of potential hosts that can be infected by these species requires some specificity, and that horizontal gene transfers can be one of the methods allowing a fast adaptation to these hosts. More interestingly, previous studies have defined groups within the pathogens or subclade P1 on the basis of virulence (outcome in patients and/or virulence in the hamster model) and phylogenomic analysis [11, 15]. Thus subgroups containing the species *L*. *interrogans*, *L*. *kirschneri* and *L*. *noguchii* on one hand and *L*. *santarosai*, *L*. *mayottensis*, *L*. *borgpetersenii*, *L*. *alexanderi* and *L*. *weilii* on the other hand are most often associated with severe infections in humans. These species diverged after a specific node of evolution (node 1 in Fig 1). The other species in subclade P1 were isolated from the environment with the exception of *L*. *alstoni* and *L*. *tipperaryensis* which were isolated from amphibians in China [47] and shrews in Ireland [33, 34], respectively. Although these other species are in the P1 subclade, they failed to induce disease or colonization in animal models like other *Leptospira* species tested in the P2, S1 and S2 subclades [15]. It is striking to note that species that diverged after node 1 harbor a lower percentage of coding sequences, and very high percentage of pseudogenes (as compared to other species) and an enrichment of genes in the category of replication, recombination and repair that includes transposase and integrase. It has been shown that mobile elements in *L*. *borgpetersenii* are likely involved in the genomic decay of the pathogen though recombination events and inactivation of genes [48]. The same study postulated that these IS-mediated events increased the dependence of *L*. *borgpetersenii* to its hosts as several genes involved in tolerance to nutrient deprivation were altered. In the present study, we also found that species that diverged after node 1 tend to be depleted in several functional categories comparatively to the other species. The mechanisms of such decay remains complicated to study given the fact that insertion sequences are one of main genomic determinants that cause contig breakages during the *de novo* assembly process [49]. Nevertheless, this phenomenon is often associated with ecological specialization and host dependence [50], which could suggest that after ongoing ecological niche switch from free living to symbiotic lifestyle (concomitant with gene expansion), this group of bacteria are now stabilizing and restricting their lifestyle in specific niches.

In conclusion, the present study, unveils the diversity of the *Leptospira* genus and the evolution of species from this genus. In the future, understanding how speciation occurs in the environment should increase our knowledge of the evolution of pathogens and acquisition of virulence factors. The increasing availability of *Leptospira* genomes that are representative of the diversity within the genus has created new opportunities for reconstructing bacterial evolution. Nevertheless, by describing several potentially infectious *Leptospira* species opens up questions about their implication in public health and diagnostic tools should be updated to take into account the new species described in the present study in order to evaluate their association with infection of both animal and humans and their role in clinical disease.

## Supporting information

S1 File ANI analyzes with all the genomes available in GenBank and the genomes of our dataset. (XLSX)

S1 Table Information on the 124 genomes investigated in this study (including accession numbers). (XLSX)

S2 Table ANI analysis with the 124 genomes of the present study. (XLSX)

S3 Table *Leptospira* species isolated in at least two different countries. (XLSX)

S4 Table Phenotypic analysis of representative species of subclade S2. (XLSX)

S1 Fig Phylogenetic tree based on the sequences of 1371 genes inferred as orthologous. The matrix represents the calculated AAI values for all the genomic sequences. The branches are colored according to their belonging to the four main subclades: P1 (red), P2 (purple), S1 (green) and S2 (blue). The bootstrap value is indicated for a single node (that corresponding to the separation between *L*. *biflexa* strain Patoc 1 and *L*. *bouyouniensis* strain 201601297) since all the others have the maximum value of 100. A circle of color, according to the legend, represents the geographical origin of each of the new species described by this study.
(TIF)

S2 Fig Phylogenetic tree based on the sequences of 1371 genes inferred as orthologous. The matrix represents the calculated POCP values for all the genomic sequences. The branches are colored according to their belonging to the four main subclades: P1 (red), P2 (purple), S1 (green) and S2 (blue). The bootstrap value is indicated for a single node (that corresponding to the separation between *L*. *biflexa* strain Patoc 1 and *L*. *bouyouniensis* strain 201601297) since all the others have the maximum value of 100. A circle of color, according to the legend, represents the geographical origin of each of the new species described by this study.
(TIF)

S3 Fig Transmission electron microscopy of representative species of subclades P1 (*L. interrogans*), P2 (*L. licerasiae*), S1 (*L. biflexa*) and S2 (*L. kobayashii*, *L. ognonensis*, and *L. ilyithenensis*). Exponential phase cultures of *L*. *kobayashii* strain E30^T^, *L*. *ilyithenensis* strain 201400974 ^T^, *L*. *ognonensis* strain 201702476^T^, *L*. *biflexa* strain Patoc1, *L*. *licerasiae* strain Var010^T^ and *L*. *interrogans* strain L495 were allowed to adsorb onto a carbon-coated copper grid. Samples were fixed with 2% glutaraldehyde, washed in distilled water and negatively stained with 4% uranyl acetate. After drying, grids were observed under a FEI Tecnai T12 Transmission Electron Microscope with an acceleration voltage of 120 kV. Electron micrographs were taken at a magnification of 2,900 on ten isolated representative cells of one strain of each described species. Measurements were done using ImageJ software.
(TIFF)

## References

[1] Costa F, Hagan JE, Calcagno J, Kane M, Torgerson P, Martinez-Silveira MS, et al Global Morbidity and Mortality of Leptospirosis: A Systematic Review. PLoS Negl Trop Dis 2015;9:e0003898 10.1371/journal.pntd.0003898 26379143 PMC4574773

[2] Ellis WA. Animal Leptospirosis In: Adler B, editor. *Leptospira* and Leptospirosis: Springer; 2014 p. 99–137.

[3] Faine SB, Adler B, Bolin C, Perolat P. *Leptospira* and leptospirosis. 2nd ed Melbourne, Australia: MediSci; 1999.

[4] Ko AI, Goarant C, Picardeau M. *Leptospira*: the dawn of the molecular genetics era for an emerging zoonotic pathogen. Nat Rev Microbiol 2009;7:736–47. 10.1038/nrmicro2208 19756012 PMC3384523

[5] Stimson AM. Note on an organism found in yellow-fever tissue. Public Health Reports (Washington). 1907;22:541.

[6] Perolat P, Chappel RJ, Adler B, Baranton G, Bulach DM, Billinghurst ML, et al *Leptospira fainei* sp. nov., isolated from pigs in Australia. Int J Syst Bacteriol. 1998;48:851–8. 10.1099/00207713-48-3-851 9734039

[7] Casanovas-Massana A, Pedra GG, Wunder EAJ, Diggle PJ, Begon M, Ko AI. Quantification of *Leptospira interrogans* Survival in Soil and Water Microcosms. Appl Environ Microbiol. 2018;84. 10.1128/AEM.00507-18 PMC6007094 29703737

[8] Andre-Fontaine G, Aviat F, Thorin C. Water borne leptospirosis: survival and preservation of the virulence of pathogenic *Leptospira* spp. in fresh water. Curr Microbiol 2015;71:136–42. 10.1007/s00284-015-0836-4 26003629

[9] Fouts DE, Matthias MA, Adhikarla H, Adler B, Berg DE, Bulach D, et al What Makes a Bacterial Species Pathogenic?: Comparative Genomic Analysis of the Genus Leptospira. PLoS Negl Trop Dis. 2016;10:e0004403 10.1371/journal.pntd.0004403 26890609 PMC4758666

[10] Picardeau M. Virulence of the zoonotic agent of leptospirosis: still terra incognita? Nat Rev Microbiol 2017;15:297–307. 10.1038/nrmicro.2017.5 28260786

[11] Xu Y, Zhu Y, Wang Y, Chang YF, Zhang Y, Jiang X, et al Whole genome sequencing revealed host adaptation-focused genomic plasticity of pathogenic *Leptospira*. Sci Rep. 2016;6:20020 10.1038/srep20020 26833181 PMC4735792

[12] Chakraborty A, Miyahara S, Villanueva SY, Saito M, Gloriani NG, Yoshida S. A novel combination of selective agents for isolation of *Leptospira* species. Microbiol Immunol. 2011;55:494–501. 10.1111/j.1348-0421.2011.00347.x 21545510

[13] Saito M, Villanueva SY, Kawamura Y, Iida KI, Tomida J, Kanemaru T, et al *Leptospira idonii* sp. nov., isolated from an environmental water in Fukuoka, Japan. Int J Syst Evol Microbiol. 2012;63:2457–62. 10.1099/ijs.0.047233-0 23203626

[14] Thibeaux R, Girault D, Bierque E, Soupé-Gilbert ME, Rettinger A, Douyère A, et al Biodiversity of Environmental Leptospira: Improving Identification and Revisiting the Diagnosis. Front Microbiol 2018;9:816 10.3389/fmicb.2018.00816 29765361 PMC5938396

[15] Thibeaux R, Iraola G, Ferrés I, Bierque E, Girault D, Soupé-Gilbert ME, et al Deciphering the unexplored *Leptospira* diversity from soils uncovers genomic evolution to virulence. Microb Genom 2018;4. 10.1099/mgen.0.000144 PMC5857368 29310748

[16] Guernier V, Allan KJ, Goarant C. Advances and challenges in barcoding pathogenic and environmental *Leptospira*. Parasitology. 2018;145:595–607. 10.1017/S0031182017001147 28716157 PMC6010154

[17] Masuzawa T, Sakakibara K, Saito M, Hidaka Y, Villanueva SYAM, Yanagihara Y, et al Characterization of *Leptospira* species isolated from soil collected in Japan. Microbiol Immunol 2018;62:55–9. 10.1111/1348-0421.12551 29105847

[18] Seemann T. Prokka: rapid prokaryotic genome annotation. Bioinformatics. 2014;15:2068–9.

[19] Contreras-Moreira B, Vinuesa P. GET_HOMOLOGUES, a versatile software package for scalable and robust microbial pangenome analysis. Appl Environ Microbiol. 2013;79:7696–701. 10.1128/AEM.02411-13 24096415 PMC3837814

[20] Katoh K, Standley DM. MAFFT multiple sequence alignment software version 7: improvements in performance and usability. Mol Biol Evol. 2013;30:772–80. 10.1093/molbev/mst010 23329690 PMC3603318

[21] Abascal F, Zardoya R, Telford MJ. TranslatorX: multiple alignment of nucleotide sequences guided by amino acid translations. Nucleic Acids Res. 2010;38:W7–13. 10.1093/nar/gkq291 20435676 PMC2896173

[22] Criscuolo A, Gribaldo S. BMGE (Block Mapping and Gathering with Entropy): a new software for selection of phylogenetic informative regions from multiple sequence alignments. BMC Evol Biol 2010;10:210 10.1186/1471-2148-10-210 20626897 PMC3017758

[23] Borowiec ML. AMAS: a fast tool for alignment manipulation and computing of summary statistics. PeerJ. 2016;4:e1660 10.7717/peerj.1660 26835189 PMC4734057

[24] Nguyen LT, Schmidt HA, vonHaeseler A, Minh BQ. IQ-TREE: a fast and effective stochastic algorithm for estimating maximum-likelihood phylogenies. Mol Biol Evol 2015;32:268–74. 10.1093/molbev/msu300 25371430 PMC4271533

[25] Hoang DT, Chernomor O, vonHaeseler A, Minh BQ, Vinh LS. UFBoot2: Improving the Ultrafast Bootstrap Approximation. Mol Biol Evol. 2018;35:518–22. 10.1093/molbev/msx281 29077904 PMC5850222

[26] Lehmann JS, Fouts DE, Haft DH, Cannella AP, Ricaldi JN, Brinkac L, et al Pathogenomic inference of virulence-associated genes in Leptospira interrogans. PLoS Negl Trop Dis. 2013;7:e2468 10.1371/journal.pntd.0002468 24098822 PMC3789758

[27] Han HJ, Wen HL, Liu JW, Qin XR, Zhao M, Wang LJ, et al Pathogenic *Leptospira* Species in Insectivorous Bats, China, 2015. Emerg Infect Dis. 2018;24:1123–6. 10.3201/eid2406.171585 29774833 PMC6004874

[28] Gurevich A, Saveliev V, Vyahhi N, Tesler G. QUAST: quality assessment tool for genome assemblies. Bioinformatics. 2013;29:1072–5. 10.1093/bioinformatics/btt086 23422339 PMC3624806

[29] Carver T, Harris SR, Berriman M, Parkhill J, McQuillan JA. Artemis: an integrated platform for visualization and analysis of high-throughput sequence-based experimental data. Bioinformatics. 2012;28:464–9. 10.1093/bioinformatics/btr703 22199388 PMC3278759

[30] Setubal JC, Reis MG, Matsunaga J, Haake DA. Lipoprotein computational prediction in spirochaetal genomes. Microbiology. 2006;152:113–21. 10.1099/mic.0.28317-0 16385121 PMC2667199

[31] Huerta-Cepas J, Forslund K, Coelho LP, Szklarczyk D, Jensen LJ, vonMering C, et al Fast Genome-Wide Functional Annotation through Orthology Assignment by eggNOG-Mapper. Mol Biol Evol. 2017;34:2115–22. 10.1093/molbev/msx148 28460117 PMC5850834

[32] Jones P, Binns D, Chang HY, Fraser M, Li W, McAnulla C, et al InterProScan 5: genome-scale protein function classification. Bioinformatics. 2014;30:1236–40. 10.1093/bioinformatics/btu031 24451626 PMC3998142

[33] Nally JE, Bayles DO, Hurley D, Fanning S, McMahon BJ, Arent Z. Complete Genome Sequence of Leptospira alstonii Serovar Room22 Strain GWTS #1. Genome Announc 2016;4:e01230–16. 10.1128/genomeA.01230-16 27834698 PMC5105091

[34] Nally JE, Arent Z, Bayles DO, Hornsby RL, Gilmore C, Regan S, et al Emerging Infectious Disease Implications of Invasive Mammalian Species: The Greater White-Toothed Shrew (Crocidura russula) Is Associated With a Novel Serovar of Pathogenic Leptospira in Ireland. PLoS Negl Trop Dis 2016;10:e0005174 10.1371/journal.pntd.0005174 27935961 PMC5147805

[35] Richter M, Rosselló-Móra R. Shifting the genomic gold standard for the prokaryotic species definition. Proc Natl Acad Sci USA. 2009;106:19126–31. 10.1073/pnas.0906412106 19855009 PMC2776425

[36] Worley J, Meng J, Allard MW, Brown EW, Timme RE. Salmonella enterica Phylogeny Based on Whole-Genome Sequencing Reveals Two New Clades and Novel Patterns of Horizontally Acquired Genetic Elements. mBio. 2018;9:e02303–18. 10.1128/mBio.02303-18 PMC6282209 30482836

[37] Qin QL, Xie BB, Zhang XY, Chen XL, Zhou BC, Zhou J, et al A proposed genus boundary for the prokaryotes based on genomic insights. J Bacteriol. 2014;196:2210–5. 10.1128/JB.01688-14 24706738 PMC4054180

[38] Eshghi A, Lourdault K, Murray GL, Bartpho T, Sermswan RW, Picardeau M, et al Leptospira interrogans catalase is required for resistance to H2O2 and for virulence. Infect Imm. 2012;80:3892–9. 10.1128/IAI.00466-12 PMC3486042 22927050

[39] He S, Gall DL, McMahon KD. "Candidatus Accumulibacter" population structure in enhanced biological phosphorus removal sludges as revealed by polyphosphate kinase genes. Appl Environ Microbiol 2007;73:5865–74. 10.1128/AEM.01207-07 17675445 PMC2074919

[40] Richert K, Brambilla E, Stackebrandt E. The phylogenetic significance of peptidoglycan types: Molecular analysis of the genera *Microbacterium* and *Aureobacterium* based upon sequence comparison of *gyrB*, *rpoB*, *recA* and *ppk* and 16S rRNA genes. Syst Appl Microbiol. 2007;30:102–8. 10.1016/j.syapm.2006.04.001 16684595

[41] Konstantinidis KT, Tiedje JM. Genomic insights that advance the species definition for prokaryotes. Proc Natl Acad Sci U S A 2005;102:2567–72. 10.1073/pnas.0409727102 15701695 PMC549018

[42] Goris J, Konstantinidis KT, Klappenbach JA, Coenye T, Vandamme P, Tiedje JM. DNA-DNA hybridization values and their relationship to whole-genome sequence similarities. Int J Syst Evol Microbiol 2007;57:81–91. 10.1099/ijs.0.64483-0 17220447

[43] Caimi K, Repetto SA, Varni V, Ruybal P. *Leptospira* species molecular epidemiology in the genomic era. Infect Genet Evol 2017;54:478–85. 10.1016/j.meegid.2017.08.013 28818623

[44] Dietrich M, Mühldorfer K, Tortosa P, Markotter W. Leptospira and Bats: Story of an Emerging Friendship. PLoS Pathog. 2015;11(e1005176). 10.1371/journal.ppat.1005176 26562435 PMC4643053

[45] Jobbins SE, Alexander KA. Evidence of *Leptospira* sp. infection among a diversity of African wildlife species: beyond the usual suspects. Trans R Soc Trop Med Hyg 2015;109:349–51. 10.1093/trstmh/trv007 25669841

[46] Rouli L, Merhej V, Fournier PE, Raoult D. The bacterial pangenome as a new tool for analysing pathogenic bacteria. New Microbes New Infect. 2015;7:72–85. 10.1016/j.nmni.2015.06.005 26442149 PMC4552756

[47] Smythe L, Adler B, Hartskeerl RA, Galloway RL, Turenne CY, Levett PN. Classification of *Leptospira* genomospecies 1, genomospecies 3, genomospecies 4 and genomospecies 5 as *Leptospira alstonii* sp. nov., *Leptospira vanthielii* sp. nov., *Leptospira terpstrae* sp. nov., *Leptospira yanagawae* sp. nov., respectively. Int J Syst Evol Microbiol. 2012;63:1859–62. 10.1099/ijs.0.047324-0 22984140

[48] Bulach DM, Zuerner RL, Wilson P, Seemann T, McGrath A, Cullen PA, et al Genome reduction in *Leptospira borgpetersenii* reflects limited transmission potential. Proc Natl Acad Sci USA. 2006;103:14560–5. 10.1073/pnas.0603979103 16973745 PMC1599999

[49] Vincent AT, Boyle B, Derome N, Charette SJ. Improvement in the DNA sequencing of genomes bearing long repeated elements. J Microbiol Methods. 2014;107:186–8. 10.1016/j.mimet.2014.10.016 25447886

[50] Siguier P, Gourbeyre E, Chandler M. Bacterial insertion sequences: their genomic impact and diversity. FEMS Microbiol Rec. 2014;38:865–91. 10.1111/1574-6976.12067 PMC7190074 24499397

