# Supplementary figures and images for: Revisiting the taxonomy and evolution of pathogenicity of the genus Leptospira through the prism of genomics
Source: PLoS Negl Trop Dis. 2019 May 23;13(5):e0007270. doi: 10.1371/journal.pntd.0007270 (PMC6532842; doi:10.1371/journal.pntd.0007270)

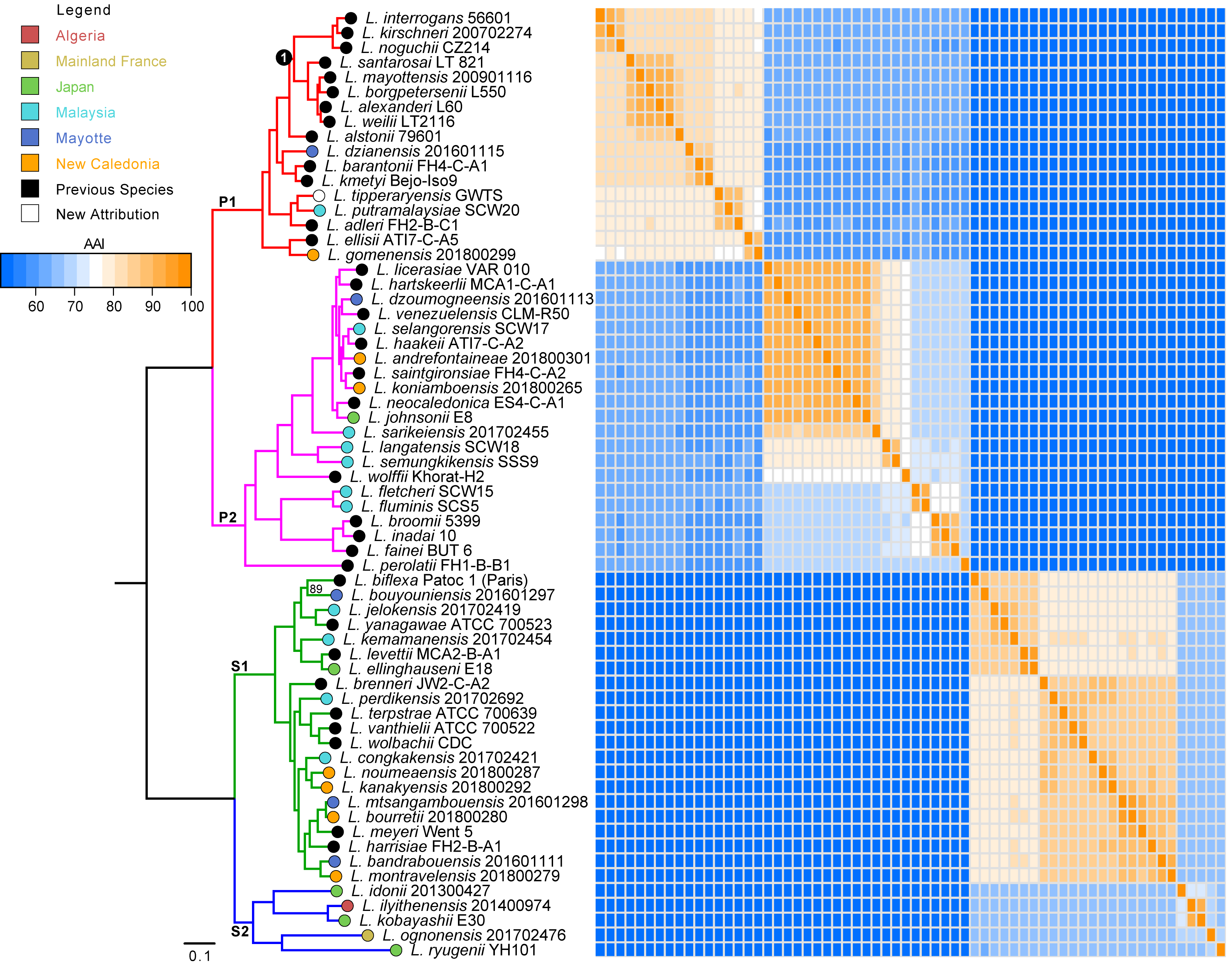

Supplement: S1 Fig — The matrix represents the calculated AAI values for all the genomic sequences. The branches are colored according to their belonging to the four main subclades: P1 (red), P2 (purple), S1 (green) and S2 (blue). The bootstrap value is indicated for a single node (that corresponding to the separation between L. biflexa strain Patoc 1 and L. bouyouniensis strain 201601297) since all the others have the maximum value of 100. A circle of color, according to the legend, represents the geographical origin of each of the new species described by this study. (TIF) [file pntd.0007270.s006.tif]

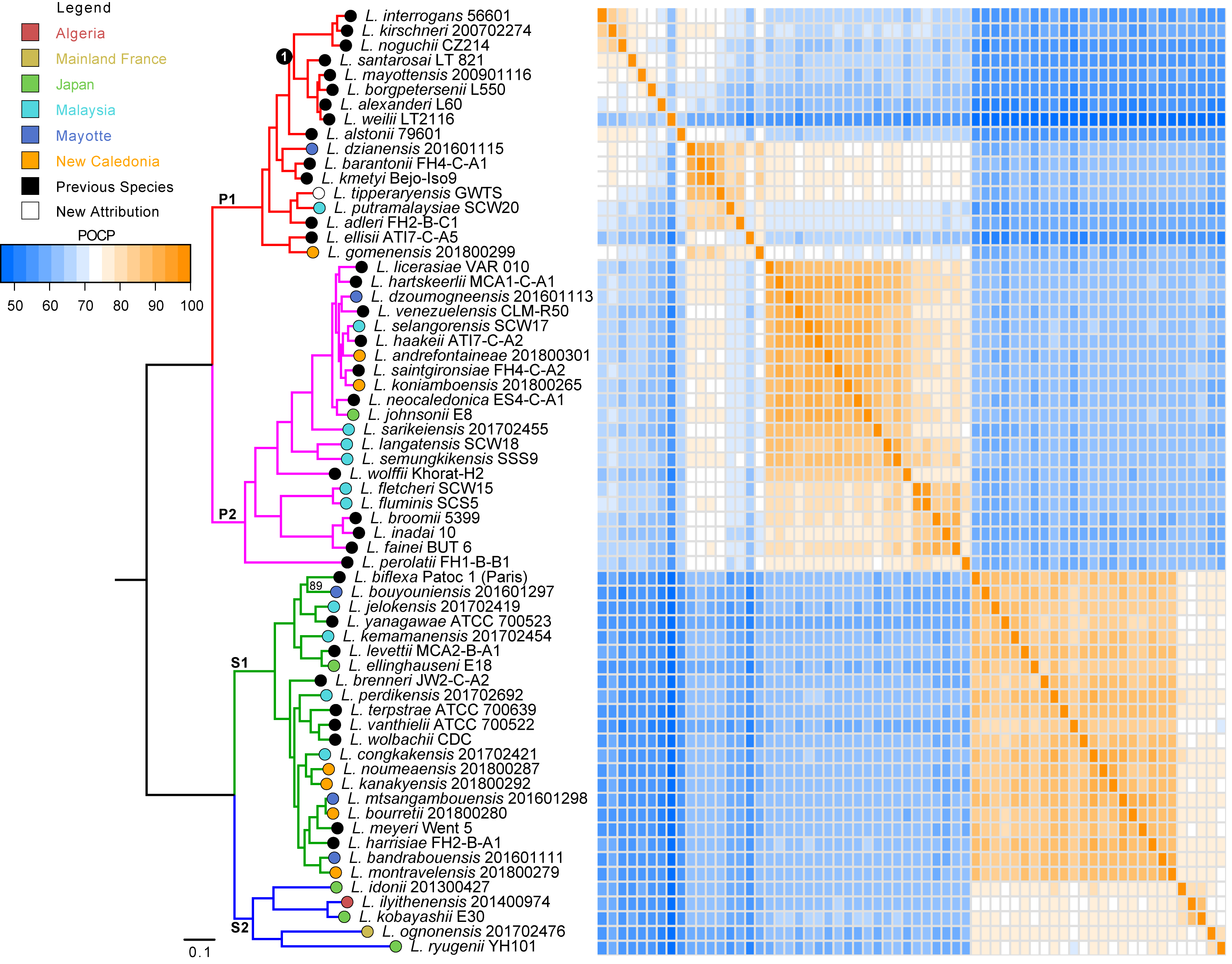

Supplement: S2 Fig — The matrix represents the calculated POCP values for all the genomic sequences. The branches are colored according to their belonging to the four main subclades: P1 (red), P2 (purple), S1 (green) and S2 (blue). The bootstrap value is indicated for a single node (that corresponding to the separation between L. biflexa strain Patoc 1 and L. bouyouniensis strain 201601297) since all the others have the maximum value of 100. A circle of color, according to the legend, represents the geographical origin of each of the new species described by this study. (TIF) [file pntd.0007270.s007.tif]

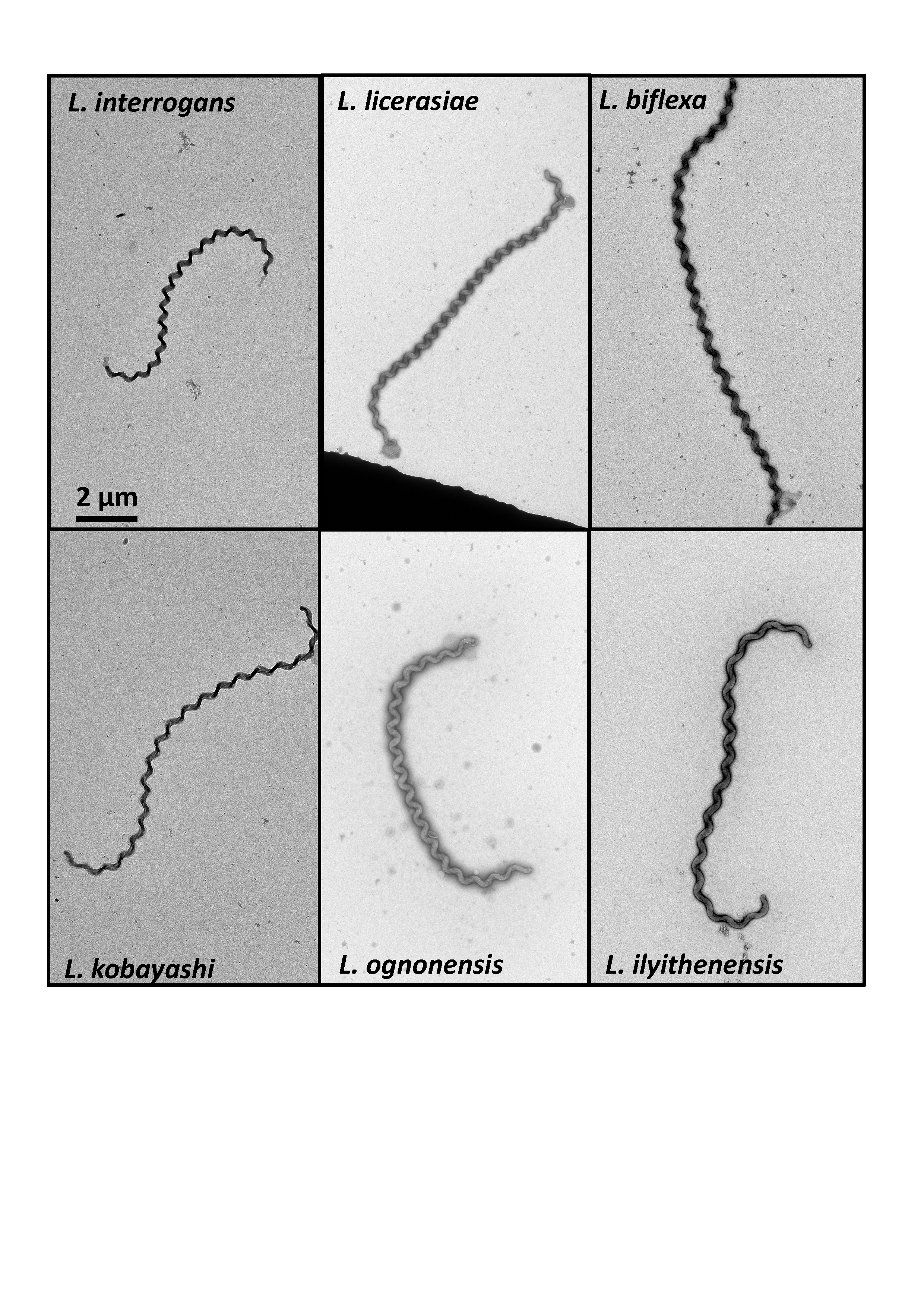

Supplement: S3 Fig — Exponential phase cultures of L. kobayashii strain E30T, L. ilyithenensis strain 201400974 T, L. ognonensis strain 201702476T, L. biflexa strain Patoc1, L. licerasiae strain Var010T and L. interrogans strain L495 were allowed to adsorb onto a carbon-coated copper grid. Samples were fixed with 2% glutaraldehyde, washed in distilled water and negatively stained with 4% uranyl acetate. After drying, grids were observed under a FEI Tecnai T12 Transmission Electron Microscope with an acceleration voltage of 120 kV. Electron micrographs were taken at a magnification of 2,900 on ten isolated representative cells of one strain of each described species. Measurements were done using ImageJ software. (TIFF) [file pntd.0007270.s008.tiff]
